# Diastereoisomers of l-proline-linked trityl-nitroxide biradicals: synthesis and effect of chiral configurations on exchange interactions†
†Electronic supplementary information (ESI) available. See DOI: 10.1039/c8sc00969d


**DOI:** 10.1039/c8sc00969d

**Published:** 2018-04-05

**Authors:** Weixiang Zhai, Yalan Feng, Huiqiang Liu, Antal Rockenbauer, Deni Mance, Shaoyong Li, Yuguang Song, Marc Baldus, Yangping Liu

**Affiliations:** a Tianjin Key Laboratory on Technologies Enabling Development of Clinical Therapeutics and Diagnostics , School of Pharmacy , Tianjin Medical University , Tianjin 300070 , P. R. China . Email: liuyangping@tmu.edu.cn ; Email: songyuguang@tmu.edu.cn; b Institute of Materials and Environmental Chemistry , Hungarian Academy of Sciences , Department of Physics , Budapest University of Technology and Economics , Budafoki ut 8 , 1111 Budapest , Hungary . Email: rockenbauer.antal@ttk.mta.hu; c NMR Spectroscopy , Bijvoet Center for Biomolecular Research , Utrecht University , 3584 CH Utrecht , The Netherlands

## Abstract

The exchange (*J*) interaction of organic biradicals is a crucial factor controlling their physiochemical properties and potential applications and can be modulated by changing the nature of the linker. In the present work, we for the first time demonstrate the effect of chiral configurations of radical parts on the *J* values of trityl-nitroxide (TN) biradicals. Four diastereoisomers (TNT_1_, TNT_2_, TNL_1_ and TNL_2_) of TN biradicals were synthesized and purified by the conjugation of a racemic (*R*/*S*) nitroxide with the racemic (*M*/*P*) trityl radical *via*l-proline. The absolute configurations of these diastereoisomers were assigned by comparing experimental and calculated electronic circular dichroism (ECD) spectra as (*M*, *S*, *S*) for TNT_1_, (*P*, *S*, *S*) for TNT_2_, (*M*, *S*, *R*) for TNL_1_ and (*P*, *S*, *R*) for TNL_2_. Electron paramagnetic resonance (EPR) results showed that the configuration of the nitroxide part instead of the trityl part is dominant in controlling the exchange interactions and the order of the *J* values at room temperature is TNT_1_ (252 G) > TNT_2_ (127 G) ≫ TNL_2_ (33 G) > TNL_1_ (14 G). Moreover, the *J* values of TNL_1_/TNL_2_ with the *S* configuration in the nitroxide part vary with temperature and the polarity of solvents due to their flexible linker, whereas the *J* values of TNT_1_/TNT_2_ are almost insensitive to these two factors due to the rigidity of their linkers. The distinct exchange interactions between TNT_1,2_ and TNL_1,2_ in the frozen state led to strongly different high-field dynamic nuclear polarization (DNP) enhancements with *ε* = 7 for TNT_1,2_ and 40 for TNL_1,2_ under 800 MHz DNP conditions.

## Abstract 2

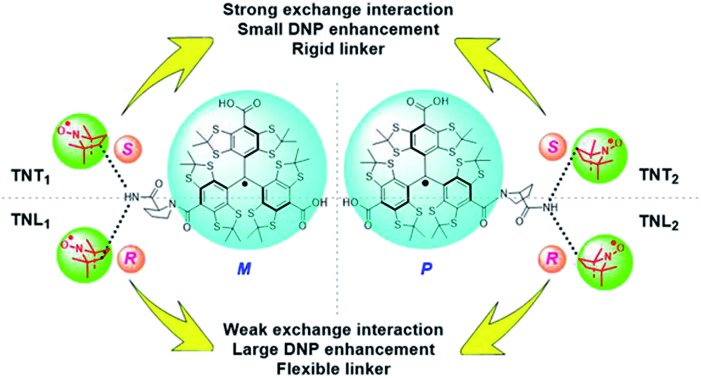
The chiral configuration of the two radical parts is a crucial factor controlling the exchange interactions and DNP properties of trityl-nitroxide biradicals.

## Introduction

Exchange-coupled biradicals have recently attracted tremendous interest owing to their unique physiochemical properties and potential applications in high-frequency dynamic nuclear polarization (DNP),^1^,^2^ molecule-based magnetism,^3^–^5^ and molecular charge transfer^6^–^8^ as well as molecular sensing.^9^,^10^ A key point in the design of new biradicals is to control the magnitude and sign of the spin–spin exchange interaction that determines their physiochemical properties and potential applications. The intramolecular exchange interaction of biradicals strongly depends on the number of chemical bonds in the linkage between the two spins, the linker conformation, and the σ/π contributions to the bonding as well as the environment (*e.g.*, temperature and solvent). This exchange interaction can be through-bond and/or through-space, and its value varies by many orders of magnitude due to different linkers between the two radical moieties.^11^,^12^ In general, for the biradicals with π-conjugated backbones, the exchange interaction is mediated more effectively *via* through-bond mechanisms. The large exchange interactions have been achieved by enhancing the degree of π-orbital overlap between the spacer and two radical moieties through a conformational constraint to enforce their coplanarity.^13^–^16^ Using donor–bridge–acceptor biradical systems, the dependence of magnetic exchange interaction on the degree of π-orbital overlap and torsional rotations between different parts has been well demonstrated.^17^–^19^ Comparatively, the exchange interactions in the biradicals with nonconjugated spacers are much weaker and can be through-bond and/or through-space, depending on the flexibility of the spacers. Recently, weakly coupled nitroxide biradicals have attracted intense attention as high-field DNP polarizing agents which boost the sensitivity of solid-state nuclear magnetic resonance spectroscopy.^20^–^27^ Nonconjugated linkers were applied in these biradicals and the rigidity and conformation of the linkers were well tailored in order to optimize the through-bond exchange and dipolar interactions and simultaneously realize the matching conditions of the EPR frequency. In addition, the supramolecular interaction with host molecules has been an effective approach to modulate the exchange interaction in nitroxide biradicals.^28^–^31^ Despite these extensive studies, to the best of our knowledge, there is no related study on the chiral effect of the linker and radical moieties on the exchange interaction of biradicals.

**Scheme 1:**
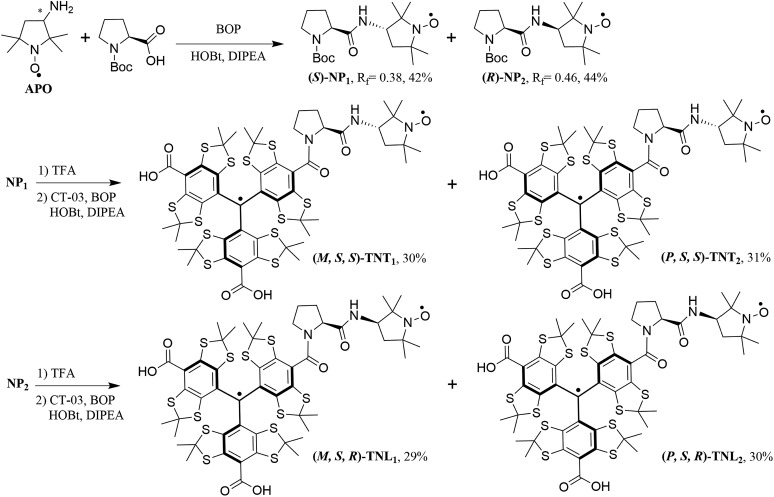
Synthesis of the four enantiomerically pure diastereoisomers of TN biradicals. *M*/*P* represents the helices of the trityl radical, while *R*/*S* indicates the configurations of l-proline and the nitroxide APO.

In the past few years, we have developed trityl-nitroxide (TN) biradicals which combine two different radical properties into unique molecules.^32^–^36^ Similar to other biradicals, the exchange interaction of TN biradicals is a key factor for their applications. We have fine-tuned the exchange interactions of TN biradicals by structural modification of the linkers^37^ as well as by supramolecular interaction with cyclodextrins.^38^ The largest DNP enhancement at a high magnetic field (18.8 T) has been achieved by using these TN biradicals as polarizing agents,^35^,^36^ in part due to the lack of nuclear depolarization.^39^ In the present work, we utilize the chiral effect of the linker and two radical parts to modulate the spin–spin exchange interaction of TN biradicals. The trityl radical has a propeller configuration which affords the right-handed (*P*) and left-handed (*M*) helices. Due to the large steric bulk of three aryl groups, the interconversion between the two enantiomeric helices is slow and they are separable at room temperature.^40^,^41^ Thus, the conjugation of the trityl radical CT-03 with the racemic mixture of the nitroxide APO through l-proline leads to four enantiomerically pure diastereoisomers (TNT_1_, TNT_2_, TNL_1_ and TNL_2_, Scheme 1) which were separated by reversed-phase (TNT_1_ and TNT_2_) or chiral (TNL_1_ and TNL_2_) HPLC. The configurations of these diastereoisomers were determined by the comparison of experimental and calculated electronic circular dichroism (ECD) spectra. The effect of the configurations of the trityl and nitroxide radicals on the exchange interactions was investigated by EPR at different temperatures and solvents. Moreover, the DNP properties of TNT_1,2_ and TNL_1,2_ were also studied at a high field (18.8 T).

## Results and discussion

### Synthesis of the four enantiomerically pure diastereoisomers

Scheme 1 shows the synthetic procedure of the four biradical diastereoisomers. The racemic mixture of the nitroxide APO was firstly coupled with *N*-Boc-l-proline to afford the nitroxide diastereoisomers NP_1_ (yield, 42%) and NP_2_ (yield, 44%) which were easily separated using column chromatography on silica gel. Then NP_1_ and NP_2_ underwent deprotection by TFA, followed by conjugation with the trityl radical CT-03 to give the biradicals TNT_1,2_ and TNL_1,2_, respectively. TNT_1,2_ displayed two close peaks (11.34 and 11.58 min) with an area ratio of 1 : 1 on reversed-phase high performance liquid chromatography (RP-HPLC, Fig. S1†) due to the *P*/*M* propeller configurations of the trityl part.^40^,^41^ Therefore, it is possible to separate the two enantiomerically pure diastereoisomers of TNT_1,2_ by semipreparative RP-HPLC and ∼2 mg of both diastereoisomers (TNT_1_ and TNT_2_) were obtained. In contrast, only one peak was observed in the RP-HPLC chromatogram of TNL_1,2_ under the same conditions (Fig. S1†). Attempts to separate the two diastereoisomers of TNL_1,2_ by optimizing the RP-HPLC conditions failed. In addition, attempts to obtain the pure diastereoisomers of TNL_1,2_ through their esterification using chiral agents such as *S*-(–)-1,1′-binaphthyl-2,2′-diol were unsuccessful. As such, the separation of TNL_1_ and TNL_2_ was achieved by analytical HPLC with a chiral stationary phase (Fig. S3†) and this separation procedure was repeated ∼50 times to afford ∼0.4 mg of TNL_1_ and ∼0.4 mg of TNL_2_. The amounts obtained for the four diastereoisomers are sufficient for subsequent ECD and EPR experiments.

### Configuration of the four biradical diastereoisomers

**Fig. 1:**
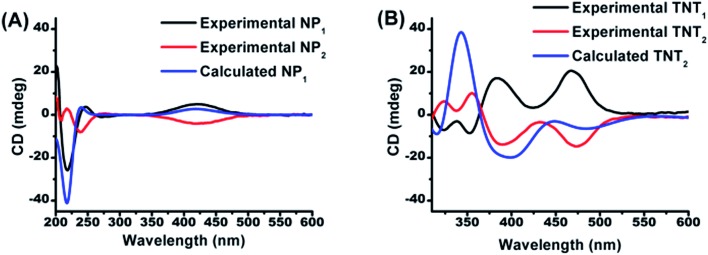
Comparison of the calculated ECD spectrum of (A) NP_1_ with the experimental ones of NP_1_ and NP_2_ and (B) of TNT_2_ with the experimental ones of TNT_1_ and TNT_2_.

In order to determine the absolute configurations of the four biradical diastereoisomers, the experimental electronic circular dichroism (ECD) spectra of NP_1_/NP_2_ and the four diastereoisomers were recorded in methanol. Meanwhile, the theoretical ECD spectra of NP_1_, TNT_2_ and TNL_2_ were also calculated using quantum chemical methods. All calculated ECD spectra with the TD-DFT method show an acceptable fitting with the corresponding experimental spectra in terms of the spectral pattern and sign of the bands with respect to the wavelength. As illustrated in Fig. 1A, NP_1_ exhibits a relatively strong ECD signal with a negative Cotton effect at 217 nm and a broad signal with a positive Cotton effect at 420 nm. Good agreement between the experimental and calculated ECD spectral patterns of NP_1_ confirms the *S* absolute configuration of the nitroxide part. Accordingly, the absolute configuration of the nitroxide part of NP_2_ is assigned as *R*.

Based on the absolute configuration of the nitroxide part, the absolute configurations of the four biradical diastereoisomers were further determined. The ECD spectrum of TNT_1_ is characteristic of the trityl radical with two strong positive Cotton effects at 384 nm and 467 nm and two relatively weak negative Cotton effects at 324 and 352 nm.^42^ Interestingly, the ECD spectra of TNT_1_ and TNT_2_ are mirror images most likely because the ECD signals of the nitroxide part in these biradical diastereoisomers are very weak and negligible as compared to the signals of the trityl part. Using TD-DFT calculations, the absolute configurations of TNT_1_ and TNT_2_ were assigned as (*M*, *S*, *S*) and (*P*, *S*, *S*), respectively. Likewise, the configurations of TNL_1_ and TNL_2_ were assigned as (*M*, *S*, *R*) and (*P*, *S*, *R*), respectively (Fig. S10†).

### Room-temperature EPR studies

**Fig. 2:**
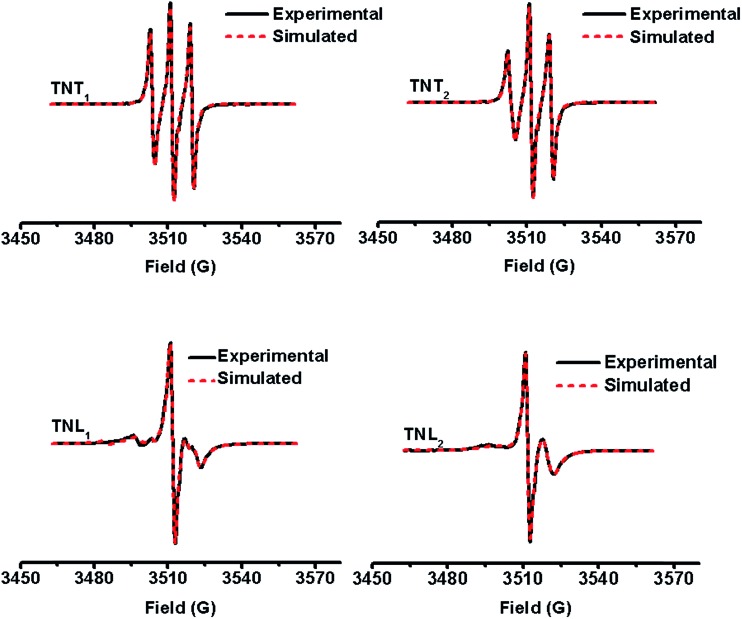
Experimental (black solid line) and simulated (red dotted line) EPR spectra of TNT_1_, TNT_2_, TNL_1_ and TNL_2_ in phosphate buffer (20 mM, pH 7.4) at room temperature.

Fig. 2 shows the EPR spectra of the four diastereoisomers in aqueous solutions at room temperature. Both TNT_1_ and TNT_2_ in phosphate buffer (PB, 20 mM, pH 7.4) exhibit well-resolved triplets with line separations of ∼8.2 G, about half the ^14^N hyperfine splitting (*α*_N_, 16.0 G) of APO. This spectral feature is characteristic of strong intramolecular exchange interaction between the two spins.^37^ Of note is that this EPR triplet pattern was only observed for the directly linked TN biradicals in previous studies.^32^–^34^,^37^ Therefore, the strong exchange interactions for TNT_1_ and TNT_2_ are unexpected given that there are multiple σ bonds (proline linker) between the two radical moieties. In contrast to TNT_1,2_, TNL_1,2_ had much more complicated and less symmetric EPR spectra with an intense central line surrounded by multiple weak peaks (TNL_1_) or one broad peak (TNL_2_) at both sides. The broad EPR peak of TNL_2_ at the left side indicates that it has a wide conformational distribution possibly due to the relatively high flexibility of the linker.^37^ Since the anisotropic hyperfine and dipolar interactions of TN biradicals are averaged out in the liquid state, the exchange interactions (*J*) can be estimated from their EPR spectra in aqueous solution. Using the well-established simulation program,^43^ the *J* values of TNL_1_ and TNL_2_ at room temperature are estimated to be 14 G and 32 G (Table 1), respectively. As expected, TNT_1_ (252 G) and TNT_2_ (128 G) have much higher *J* values than TNT_1,2_. Interestingly, the *β* and *γ* relaxation parameters in the line width formula of the nitrogen hyperfine pattern obtained for TNL_1,2_ are larger than those of TNT_1,2_, indicating that the molecular rotation of TNL_1,2_ is slower than that of TNT_1,2_. The slow molecular rotation further demonstrates that TNL_1,2_ has loose geometries where the effective rotational radius is rather large and the two radical moieties are far away from each other. This spatial separation of the two radical moieties in TNL_1,2_ may further explain why they have small *J* values as compared to TNT_1,2_. Taken together, the much stronger exchange interaction of TNT_1,2_ than TNL_1,2_ suggests that the *R*/*S* configurations of the nitroxide part play more important roles in modulating their exchange interactions than the *M*/*P* helices of the trityl part. This is unsurprising since the molecular structure of the trityl radical has high symmetry. Thus, it can also be deduced that the relative geometric conformation between the trityl part and the proline linker is similar for TNT_1_/TNT_2_ or TNL_1_/TNL_2_. To verify this hypothesis, the four diastereoisomers of TN biradicals were reduced with ascorbate to the corresponding trityl monoradicals and their EPR spectra were recorded. As shown in Fig. S5,† the four trityl monoradicals had almost identical and partially overlapped triplet signals under anaerobic conditions due to the hyperfine splitting (*α*_N_, 0.18 G) of the nitrogen from the proline linker. The identical *α*_N_ value confirms that the relative geometric conformation between the trityl part and the proline linker is the same for the four monoradicals and the *M*/*P* helices of the trityl part have a minor effect on their exchange interactions.

**Table 1:** Exchange (*J*, G) and dipolar (*D*, G) interactions of TN biradicals at room temperature (298 K) and low temperature (∼220 K)

Biradical	*J* (RT)	*J* (LT)	*D*
TNT_1_	252	196	9
TNT_2_	127	172	11
TNL_1_	14	5.4	8
TNL_2_	33	6.3	8

### Effect of temperature on the exchange interactions

**Fig. 3:**
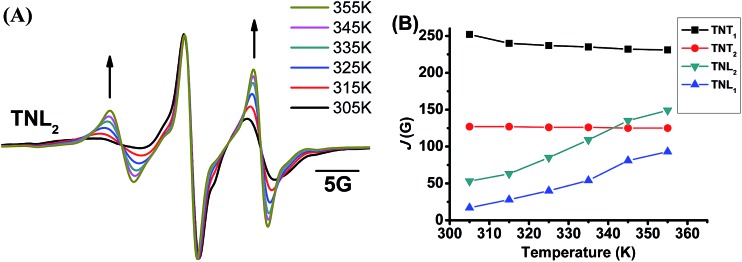
(A) Normalized EPR spectra of TNL_2_ in phosphate buffer (PB, 20 mM, pH 7.4) at different temperatures. (B) *J* values of the four biradical diastereoisomers as a function of temperature (305–355 K).

The through-space exchange interaction of the biradicals with flexible linkers can be enhanced at high temperature due to the rapid rotation of the chemical bonds in the linkers which increases the collisional frequencies of the two radical parts. As mentioned above, TNL_2_ has a flexible linker as evidenced by its broad EPR low-field peak. To further investigate the effect of the linker flexibility of the four diastereoisomers on their exchange interactions, their EPR spectra were recorded at high temperatures in the range of 305 K to 355 K. As shown in Fig. 3A, the EPR spectra of TNL_2_ exhibited outstanding changes with temperature with two relatively stronger peaks at both sides than the central peak at high temperature. Similar results were also observed for TNL_1_. However, the EPR spectra of the two diastereoisomers TNT_1,2_ at 355 K did not exhibit a significant change except for slightly narrow linewidths (Fig. S8†). Spectral simulation revealed that the *J* values of TNL_1_ and TNL_2_ increased with temperature from 17 G (305 K) to 93 G (355 K) and 53 G (305 K) to 149 G (355 K), respectively. The positive response of *J* values with temperature for TNL_1,2_ can be explained by their flexible linkers which allow for fast rotation of the chemical bonds in the linker, increase the collisional frequencies of the two radical moieties and further enhance the through-space exchange interactions.^44^ In contrast, the EPR spectra of TNT_1,2_ had no noticeable changes at different temperatures and their *J* values kept constant throughout the temperature range studied, implying that these two biradical diastereoisomers have rigid linkers (Fig. S8†). Therefore, the configuration of the nitroxide part plays a critical role in modulating the flexibility of the linkers and further the dependence of the exchange interactions on temperature.

### Solvent effect on the exchange interactions

Solvents have an important influence on the exchange interaction of biradicals by changing the population of the conformations or collisional frequencies of their two radical parts. Thus, the solvent effect on the exchange interactions of the four biradical diastereoisomers was also investigated in seven solvents (PB, MeOH, DMF, acetonitrile, acetone, THF and dioxane) at room temperature. EPR spectra were recorded (Fig. S9†) under anaerobic conditions where the potential effect of oxygen concentration on the EPR linewidth can be eliminated. These four diastereoisomers, in general, have relatively low magnitudes of exchange interactions in polar solvents (Table 2). For example, the *J* values of the two diastereoisomers of TNL_1,2_ exhibited a negative correlation with the solvent polarities. As for TNT_1,2_, strong exchange interactions were consistently observed with no marked difference in all organic solvents. Considering the large *J* values of the four diastereoisomers in viscous dioxane (1.54), enhanced exchange interactions in organic solvents most likely arises from the conformational conversion instead of the increased intramolecular collisional frequencies between the two radical parts. Since TNT_1,2_ have very rigid linkers, their conformations are strongly solidified and little dependence of their exchange interactions on solvents is expected.

**Table 2:** Exchange interaction (*J*, G) of TNT_1,2_ and TNL_1,2_ in various solvents at room temperature^*a*^

Biradical	PB	DMF	MeOH	ACN	DMK	THF	Diox
TNT_1_	252	301	342	322	310	313	373
TNT_2_	127	303	308	356	378	380	383
TNL_1_	14	58	58	209	227	281	351
TNL_2_	33	110	101	233	276	282	293

^*a*^PB, phosphate buffer. DMF, *N*,*N*-dimethylformamide. MeOH, methanol. ACN, acetonitrile. DMK, acetone. THF, tetrahydrofuran. Diox, 1,4-dioxane.

### Solid-state EPR studies and spectral simulation

**Fig. 4:**
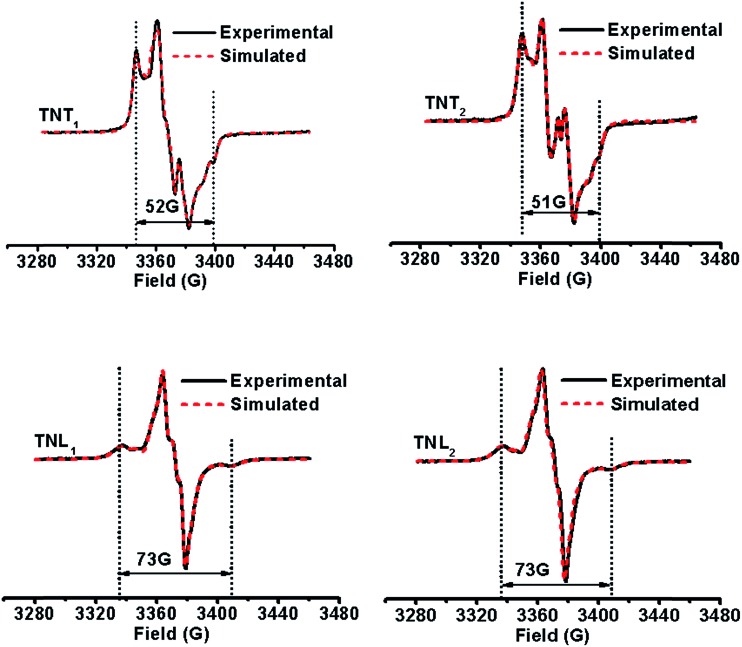
Experimental (black solid line) and simulated (red dotted line) EPR spectra of TNT_1_, TNT_2_, TNL_1_ and TNL_2_ in glycerol/water (v/v, 60/40) at low temperatures (∼220 K).

It has been demonstrated that the magnitudes of both dipolar and exchange interactions are very critical for the applications of biradicals in DNP which is carried out at low temperature. For this reason, the estimation of these two parameters from the frozen-solution spectra was attempted. Fig. 4 shows the EPR spectra of the four diastereoisomeric biradicals in a 60 : 40 (v/v) glycerol/H_2_O glass-forming solution at low temperature (∼220 K). At the first glimpse, the two diastereoisomers of TNT_1,2_ have strong exchange interactions as reflected from small overall separations between the outermost lines (*i.e.*, 52 G for TNT_1_ and 51 G for TNT_2_) in their EPR spectra which are much smaller than 2Azz (∼70 G) of the nitroxide radical. Comparatively, the exchange interactions of the two diastereoisomers of TNL_1,2_ are identical and quite weak since there is a large overall separation of 73 G between the outermost lines in their EPR spectra. In order to reliably determine *D* and *J* values from the frozen solution spectra, especially when the *J* coupling is smaller than the dipolar and hyperfine interactions, we modified our previous EPR simulation program (ROKI/EPR)^37^ by taking into account the anisotropic dipolar interaction besides *g* and hyperfine tensors. Note that this new program (called ROKI/DNP) enables the precise measurement of *D* and *J* values even determining the sign of *J*. Furthermore, it can also determine two polar angles which define the orientation of the linker in the principal direction of the nitroxide moiety. If the spectra are recorded at a high resonance frequency, the three Euler angles can be estimated giving the relative orientation of the principal axes of *g* and hyperfine tensors between the nitroxide and the trityl moieties (see more details in the Experimental section). As shown in Table 1, the order of the *J* values for the four biradical diastereoisomers in the frozen state is TNT_1_ (196 G) ∼ TNT_2_ (172 G) ≫ TNL_2_ (6.3 G) ∼ TNL_1_ (5.4 G). This trend is similar to the results obtained at room temperature. As mentioned above, the flexible linkers in TNL_1,2_ allow for the spatial proximity of the two radical parts and their exchange interactions are mostly based on a though-space mechanism above room temperature. In the frozen state, their molecular conformations were immobilized and their through-space exchange interactions were inhibited. Thus, the very weak exchange interactions observed for TNL_1,2_ in the frozen state are most likely due to the through-bond mechanism. The close *J* values of TNL_1_ and TNL_2_ indicate that they have similar preferential conformation(s) at low temperature. The similar conformations of TNL_1,2_ in the frozen state were further confirmed by their identical dipolar interactions (*D* = 8 G) which indicate the same distances between the two spins in TNL_1,2_. Comparatively, the exchange interactions of TNT_1,2_ did not significantly vary with temperature and had similar *J* values at ∼220 K and room temperature. These temperature-insensitive exchange interactions are due to the rigid linkers in TNT_1,2_ which prevent the rotation of the chemical bonds in the linker and solidify their molecular conformations.

### Dynamic nuclear polarization studies

**Fig. 5:**
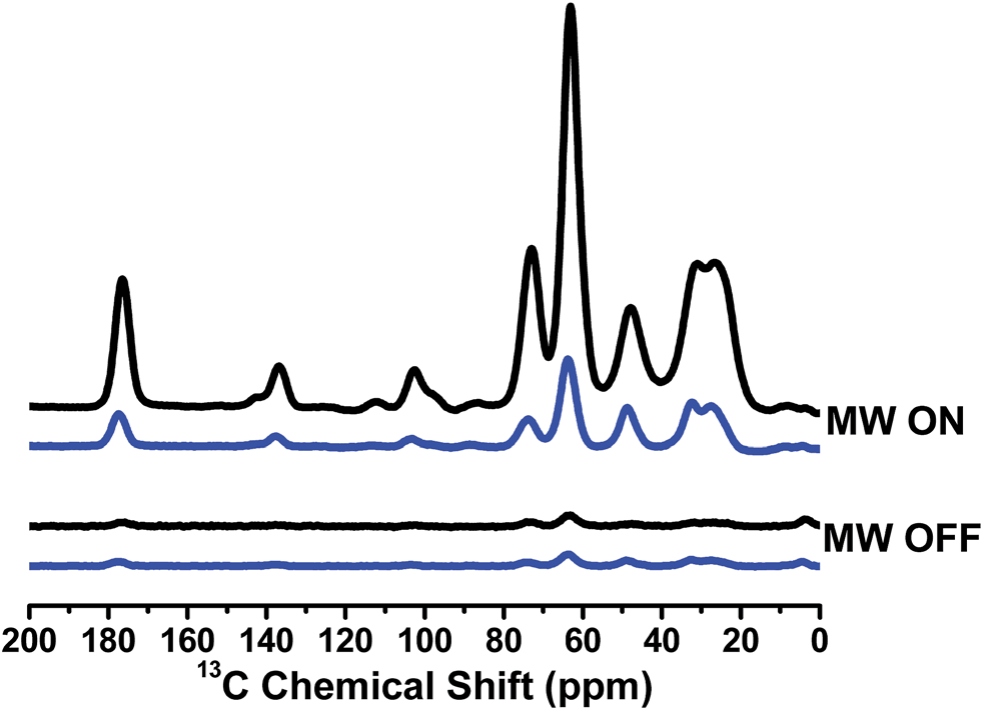
^13^C CP-MAS spectra of TNT_1,2_ (blue) and TNL_1,2_ (black) measured on the sample containing 0.25 M U–^13^C–^15^N proline with 10 mM TNT_1,2_ or TNL_1,2_ in [D_8_]glycerol/D_2_O/H_2_O (60/30/10, volume ratio) at 18.8 T (800 MHz). The measurements were carried out at 100 K with a MAS rate of 8 kHz.

Since TN biradicals are very promising DNP polarizing agents especially at a high field,^35^,^36^ the DNP performances of TNT_1,2_ and TNL_1,2_ were also evaluated on an 800 MHz/527 GHz DNP/MAS solid-state NMR system.^45^ The enhancements of ^13^C NMR signals of ^13^C–^15^N proline were measured using TNT_1,2_ and TNL_1,2_ as polarizing agents. As shown in Fig. 5, TNL_1,2_ exhibits an over 40-fold signal enhancement, whereas TNT_1,2_ has only a 7-fold enhancement. The distinct enhancements imply that the nitroxide chirality and thus the amplitude of the exchange interactions significantly influence the DNP properties of TNT_1,2_ and TNL_1,2_. Our present study experimentally verified the adverse effect of the too strong exchange interactions of TNT_1,2_ on its DNP enhancement although a similar result was theoretically suggested in a previous study.^46^ In addition, the exchange interaction may affect the electron spin relaxation of biradicals^47^ that has been shown to be a crucial factor controlling the DNP properties of nitroxide biradicals.^48^,^49^ Thus, the effect of the exchange interaction is multiple. Detailed theoretical simulations may quantitatively describe the effect of the exchange interactions on the DNP properties of TN biradicals.^39^,^50^ On the other hand, the 40-fold signal enhancement of TNL_1,2_ is somewhat lower than that previously seen for TEMTriPol-1 (*ε* = 65 for ^13^C-urea) which differs from TNL_1,2_ only by the linker and has an exchange coupling of 17 G determined by the new simulation program, which was reported to be 26 G in a previous paper.^36^ Since TNL_1,2_ (*J* ∼ 6 G) has weaker exchange interaction than TEMTriPol-1 (*J* = 17 G), an optimal *J* value for TN biradicals may exist that is large enough for highly efficient polarization transfer but is so small as not to interfere with the frequency matching required for DNP.^51^

## Conclusions

We synthesized four enantiomerically pure diastereoisomers (TNT_1_, TNT_2_, TNL_1_ and TNL_2_) of TN biradicals with different chiral configurations in the nitroxide and trityl parts. The chiral configuration of the nitroxide part plays important roles in modulating the exchange interactions of these diastereoisomers and their dependence on temperature and solvents due to the different flexibilities of the linkers. The diastereoisomeric mixture TNL_1,2_ with a small and similar *J* value (∼6 G) has a much higher DNP enhancement (*ε* = 40) than TNT_1,2_ (*J* > 190 G, *ε* = 7), accounting for the adverse effect of too strong exchange interactions on the DNP enhancement.^36^ This study demonstrates that the chiral configuration of the radical part is an effective factor modulating the exchange interaction of TN biradicals and this strategy can be used to develop new biradicals with improved DNP properties.

## Experimental section

### General information

All reactions were carried out under an argon atmosphere. Dichloromethane (CH_2_Cl_2_) was redistilled with CaH_2_ and dimethylformamide (DMF) were passed through a column of molecular sieves. Boc-l-proline, 1-hydroxybenzotriazole (HOBt), (benzotriazol-1-yloxy)tris(dimethylamino)phosphoniumhexafluoro-phosphate (BOP), *N*,*N*-diisopropylethyl-amine (DIPEA), 2,2,5,5-tetramethyl-3-amino-pyrrolidine-1-oxyl free radical (APO) and ascorbic acid were purchased and used without purification. CT-03 was prepared according to a previously reported method.^52^ Thin layer chromatography was performed on 0.25 mm silica gel plates. Flash column chromatography was employed using silica gel with a 200–300 mesh. Thin layer chromatography plates were visualized by exposure to UV light. High-resolution mass spectrometry was carried out in methanol employing electrospray ionization (ESI) methods. High resolution mass spectrometry (HRMS) analyses were performed on a LCMS-IT-TOF Shimadzu liquid chromatograph mass spectrometer. EPR measurements were carried out on a Bruker EMX-plus X-band spectrometer. ECD spectra were recorded on a Jasco J715 spectropolarimeter (Jasco Corporation, Tokyo, Japan). Analytical HPLC was carried out on an Agilent 1100 equipped with a G1315B DAD detector and G1311A pump. Semipreparative HPLC was carried out on an SSI 1500 equipped with a UV/vis detector and versa pump.

### Synthesis

#### NP_1_ and NP_2_

To a solution of Boc-l-proline (65 mg, 0.3 mmol), HOBt (111 mg, 0.82 mmol) and DIPEA (0.232 mL, 1.37 mmol) in CH_2_Cl_2_ (3 mL), BOP (145 mg, 0.33 mmol) was added. The resulting solution was stirred at ambient temperature for 0.5 h. Then, a solution of the nitroxide APO (43 mg, 0.27 mmol) in CH_2_Cl_2_ (1 mL) was added and the reaction mixture was stirred at 25 °C for another 3 h. CH_2_Cl_2_ (15 mL) was added and the organic layer was washed successively with 6% citric acid (10 mL), saturated solution of NaHCO_3_ (10 mL) and brine (10 mL). The organic layer was dried over anhydrous sodium sulfate, filtered and concentrated *in vacuo*. The crude residue was purified by flash column chromatography on silica gel using EtOAc/petroleum ether (1 : 2) as an eluent to give two diastereoisomers NP_1_ (41 mg, 42% yield) as a yellow oil and NP_2_ (43 mg, 44% yield) as a yellow solid. NP_1_, MS (ESI, [M + H]^+^, *m*/*z*): 355.2 (measured), 355.2 (calculated); ([M + Na]^+^, *m*/*z*): 377.2 (measured), 377.2 (calculated). EPR: *α*_N_ = 15.7 G in MeOH/H_2_O (v/v, 1 : 2) and 14.5 G in CH_2_Cl_2_. NP_2_, MS (ESI, [M + H]^+^, *m*/*z*): 355.2 (measured), 355.2 (calculated); (ESI, [M + Na]^+^, *m*/*z*): 377.3 (measured), 377.2 (calculated). EPR: *α*_N_ = 15.7 G in MeOH/H_2_O (v/v, 1/2) and 14.5 G in CH_2_Cl_2_.

#### TNT_1_ and TNT_2_

A solution of NP_1_ (38 mg, 0.10 mmol) in CH_2_Cl_2_ (1 mL) was treated with trifluoroacetic acid (TFA, 1 mL) and the resulting solution was stirred at 25 °C for 2 h. After removing the solvents under *vacuo*, the residue was redissolved in DMF (2 mL). To a solution of CT-03 (53 mg, 0.053 mmol), HOBt (22 mg, 0.16 mmol) and DIPEA (90 μL, 0.53 mmol) in DMF (3 mL), BOP (24 mg, 0.05 mmol) was added. The resulting solution was stirred at 24 °C for 0.5 h and then mixed with the solution of the deprotected NP_1_ in DMF (2 mL). After stirring at 25 °C for another 18 h, the reaction mixture was poured into EtOAc (10 mL) and 1 M HCl (10 mL). The organic layer was separated and washed with brine (2 × 10 mL), dried over anhydrous sodium sulfate, filtered and concentrated *in vacuo*. The resulting residue was dissolved in phosphate buffer (0.2 M, pH 7.4) and purified by column chromatography on a reversed-phase C18 using water followed by 0–40% MeOH in H_2_O as eluents to give the diastereoisomeric mixture TNT_1,2_ (43 mg, 61%). HPLC analysis of the mixture on the reversed-phase C18 column showed that there were two close peaks with the retention times of 11.34 (TNT_1_) and 11.58 min (TNT_2_) (Fig. S1†). Subsequently, TNT_1_ and TNT_2_ were separated by semipreparative HPLC on a Grace Apollo column C18 (5 μm, 150 mm × 22 mm). Column temperature: 25 °C, UV detection at 254 nm, flow rate: 5 mL min^–1^, and eluent: acetonitrile/NH_4_OAc (20 mM, pH 6.8), 30%/70% (0 min)–50%/50% (20 min). An amount of ∼2 mg was obtained for each diastereoisomer. TNT_1_, HRMS (ESI, [M – 2H]^2–^, *m*/*z*): 617.5496 (measured), 617.0540 (calculated). TNT_2_, HRMS (ESI, [M – 2H]^2–^, *m*/*z*): 617.5486 (measured), 617.0540 (calculated).

#### TNL_1_ and TNL_2_

A similar procedure for the synthesis and purification of TNT_1_ and TNT_2_ was applied. The diastereoisomeric mixture TNL_1,2_ (40 mg) was obtained from NP_2_ (40 mg, 0.11 mmol) and CT-03 (55 mg, 0.055 mmol) in a yield of 59%. Attempts to separate TNL_1_ and TNL_2_ from the mixture by reversed-phase C18 HPLC failed. Fortunately, TNL_1_ and TNL_2_ were appropriately separated on a chiral HPLC CHIRALPAK® IG column (5 μm, 1.6 mm × 250 mm) with the retention times of 7.89 and 10.65 min for TNL_1_ and TNL_2_, respectively (Fig. S2†). Column temperature: 25 °C, UV detection at 254 nm, flow rate: 1 mL min^–1^, and eluent: ethanol/*n*-hexane 25%/75% (0.1% AcOH). Repeating this separation procedure (∼50 times) afforded small amounts (∼0.4 mg) of these two diastereoisomers. TNL_1_, HRMS (ESI, [M – 2H]^2–^, *m*/*z*): 617.5497 (measured), 617.0540 (calculated). TNL_2_, HRMS (ESI, [M – 2H]^2–^, *m*/*z*): 617.5482 (measured), 617.0540 (calculated).

### Reduction of the four biradical diastereoisomers by ascorbic acid

Each diastereoisomer (50 μM) was mixed with ascorbic acid (2 mM) in PBS (20 mM, pH 7.4). After the reaction was completed, the resulting solution was transferred into a capillary tube and EPR spectra were recorded using the following EPR parameters: microwave power, 0.5 mW; modulation amplitude, 0.03 G.

### EPR spectroscopy

EPR measurements were carried out on a Bruker EMX-plus X-band spectrometer at room temperature (298 K) or low temperature (∼220 K). General instrumental settings were as follows: modulation frequency, 100 kHz; microwave power, 10 mW; and modulation amplitude, 1 G for room temperature and 2 G for low temperature. Measurements were performed in 50 μL capillary tubes. Spectral simulation was performed by using the program developed by Professor Rockenbauer.^43^ In this study, the exchange, dipolar and hyperfine couplings are given in Gauss units which can be converted into cm^–1^ by multiplying with *g* × 4.6686 × 10^–5^, where *g* is the respective Zeeman factor. EPR measurements under anaerobic conditions were carried out using a gas-permeable Teflon tube (i.d. = 0.8 mm). Briefly, the sample solution was transferred to the tube which was then sealed at both ends. The sealed sample was placed inside a quartz EPR tube with open ends. Argon gas was allowed to bleed into the EPR tube, and then an EPR spectrum was recorded.

### EPR simulation

The EPR spectra are computed by using a perturbation solution of the biradical spin Hamiltonian:*H*_SH_ = *Hĝ*_1_*S*_1_*μ*_B_ + *Hĝ*_2_*S*_2_*μ*_B_ + *JS*_1_*S*_2_ + *D*′′(2*S*_*z*1_*S*_*z*2_ – *S*_*x*1_*S*_*x*2_ – *S*_*y*1_*S*_*y*2_) + *S*_1_*Â*_1_*I*_1_ + *S*_2_*Â*_2_*I*_2_

The computations are carried out in three different frames:

(1) The (*x*, *y*, *z*) reference frame is chosen as the principal axis of the *ĝ*_1_ Zeeman and *Â*_1_ hyperfine tensor of the nitroxide moiety. The orientation of the magnetic field is given by the *θ* and *φ* polar angles and defined in the above frame.*H*_*x*_ = *H*_0_ sin *θ* cos *φ*, *H*_*y*_ = *H*_0_ sin *θ* sin *φ*, *H*_*z*_ = *H*_0_ cos *θ*

(2) The slightly anisotropic *ĝ*_2_ tensor and a small *Â*_2_ hyperfine tensor of the trityl are defined in the (*x*′, *y*′, *z*′) frame. The relative orientation of the two frames is characterized by the three Euler angles *α*, *β* and *γ*:
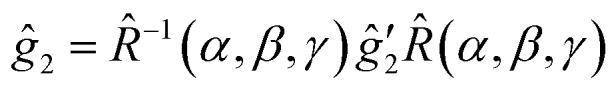


The same transformation defines the relationship of the hyperfine tensors *Â*_1_ and *Â*_2_.

(3) The dipolar interaction *D*′′ is defined in a third frame, where the *z*′′ axis shows the direction of the linker and its orientation is given by two polar angles *ξ* and *ζ*. A perturbation procedure is applied in the case when the Zeeman term is large compared to the exchange, dipolar and hyperfine interactions. Then in the Zeeman and hyperfine terms, only the *z* component of the electron and nuclear spin remains:*H*_Z_ = *H*_0_*g*_1_(*θ*_1_, *φ*_1_)*μ*_B_*S*_*z*1_ + *H*_0_*g*_2_(*θ*_2_, *φ*_2_)*μ*_B_*S*_*z*2_*H*_HF_ = *A*_1_(*θ*_1_, *φ*_1_)*S*_*z*1_*I*_*z*1_ + *A*_2_(*θ*_2_, *φ*_2_)*S*_*z*2_*I*_*z*2_

Here the *g*-factor is
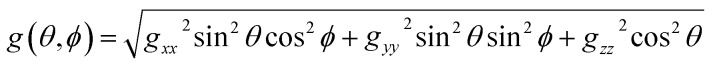


The angular dependence of the hyperfine constant is
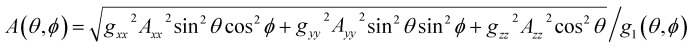


The angle dependence of the dipolar interaction is given as*D* = *D*′′/2(1 – 3 cos^2^ *δ*)

The *δ* angle between the magnetic field and the *z*′′ axis can be expressed by the polar angles *ξ* and *ζ*:cos *δ* = sin *θ* sin *ξ* cos *φ* cos *ζ* + sin *θ* sin *ξ* sin *φ* sin *ζ* + cos *θ* cos *ξ*

The biradicals have four energy levels that can be assigned to the *S* = 1 triplet and *S* = 0 singlet state. Both the exchange and dipolar terms can mix the *M*_S_ = 0 states of the *S* = 1 triplet and *S* = 0 singlet:*H*_Exch_ + *H*_Dip_ = *S*_*z*1_*S*_*z*2_(2*D* + *J*) + 1/2(*S*_+1_*S*_–2_ + *S*_–1_*S*_+2_)(*J* – *D*)

The diagonal elements in the |*S*, *M*_S_ basis are1, 1|*H*_Exch_ + *H*_Dip_|1, 1 = 1, –1|*H*_Exch_ + *H*_Dip_|1, –1 = *J*/4 + *D*/21, 0|*H*_Exch_ + *H*_Dip_|1, 0 = *J*/4 – *D*0, 0|*H*_Exch_ + *H*_Dip_|0, 0 = –3*J*/4while the off-diagonal elements are zero.

The Zeeman and hyperfine interactions have only diagonal elements in the |*S*, *M*_S_, *M*_I1_, *M*_I2_ basis if *M*_S_ = 1 or –1:1, 1|*H*_Z_ + *H*_HF_|1, 1 = –1, –1|*H*_Z_ + *H*_HF_|1, –1 = 1/2[(*g*_1_ + *g*_2_)*μ*_B_*H*_0_ + *A*_1_*M*_I1_ + *A*_2_*M*_I2_]

For the *M*_S_ = 0 state, these interactions give zero diagonal elements, but in this case the off-diagonal elements can be non-zero:1, 0|*H*_Z_ + *H*_HF_|0, 0 = 1/2[(*g*_1_ – *g*_2_)*μ*_B_*H*_0_ + *A*_1_*M*_I1_ – *A*_2_*M*_I2_]

Since the |1, 1 and |1, –1 triplet states are well separated from the central states, the eigenvalues can be obtained directly from the diagonal elements. We introduce the indices *i* = 1 and 4 for the highest |1, 1 and lowest |1, –1 states, respectively. Furthermore *i* = 2 and 3 denote the two central states with *M*_S_ = 0.*E*_1,*M*_I1_,*M*_I2__ = –*E*_4,*M*_I1_,*M*_I2__ = *J*/4 + *D*/2 + 1/2(*g*_1_ + *g*_2_)*μ*_B_*H*_0_ + 1/2(*A*_1_*M*_I1_ + *A*_2_*M*_I2_)

The mixing of |1, 0 and |0, 0 states can be obtained by the solution of the secular equation:|2 = *p*_1_|1, 0 + *p*_2_|0, 0|3 = *p*_2_|1, 0 – *p*_1_|0, 0

The mixing coefficients can be expressed by the factor *R*:*R*^2^ = (*J* – *D*)^2^ + ((*g*_1_ – *g*_2_)*μ*_B_*H*_0_ + *A*_1_*M*_I1_ – *A*_2_*M*_I2_)^2^*p*_1_^2^ = 1/2|*R* + *J* – *D*|/*R*, *p*_2_^2^ = 1/2|*R* – *J* + *D*|/*R*

It is worth noting that the transition probability and energy depend on the relative sign of *D* and *J*, which offers a possibility to decide from the frozen solution EPR spectra whether the Heisenberg exchange is ferro- or anti-ferromagnetic. Then, the energy expressions for the central states are*E*_*i*,*M*_I1_,*M*_I2__ = –*J*/4 – *D*/2 ± *R*/2, *i* = 2, 3.

Due to the triplet–singlet mixing, four transitions can take place in the whole *M*_I1_, *M*_I2_ subspace. The two resonances have the transition probability*W*_12_ = *W*_24_ = |1|*S*_+_|2|^2^ = 2*p*_1_^2^

The respective frequency is*ℏω*_12 or 24_ = 1/2(*g*_1_ + *g*_2_)*μ*_B_*H*_0_ + 1/2(*A*_1_*M*_I1_ + *A*_2_*M*_I2_) ± (*J*/2 + *D* – *R*/2).

The above resonances can be called “allowed”, since their intensities are larger than 1.0 and the line separations are smaller than *J*. The other pair of resonances has the transition probability*W*_13_ = *W*_34_ = |1|*S*_+_|3|^2^ = 2*p*_2_^2^

This transition can be considered as “forbidden” due to the smaller transition probability: 2*p*_2_^2^ < 1.0 and the frequency spread can be extended in a broad range comparable with the value of *J*:*ℏω*_13 or 34_ = 1/2(*g*_1_ + *g*_2_)*μ*_B_*H*_0_ + 1/2(*A*_1_*M*_I1_ + *A*_2_*M*_I2_) ± (*J*/2 + *D* + *R*/2)

For a fixed *ω*_EPR_ microwave frequency the above relationships allow the determination of the corresponding four resonance fields *H*_*i*,*M*_I1_,*M*_I2__(*θ*, *φ*), where the *i* index denotes the four transitions.

The ROKI/DNP program can use either Lorentzian or Gaussian, or mixed line shapes *f*(*H*) and the powder pattern can be obtained by integrating over the angles *θ* and *φ*:
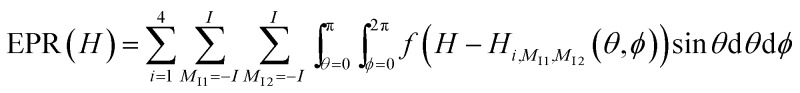


All parameters can be optimized to achieve the best fit of the experimental spectra; the method of optimization is the same as that described in the ROKI/EPR program.^43^ Due to the large number of optimized parameters ambiguity can occur. This problem can be reduced by recording the frozen solution spectra at different resonance frequencies. In X band spectra the determination of the three Euler angles is rather problematic due to the small g anisotropy of the trityl moiety. The polar angles, however, are more reliable since the nitroxide part has a large Zeeman and hyperfine anisotropy.

### DNP/ssNMR spectroscopy of the diastereoisomeric mixtures TNT_1,2_ and TNL_1,2_

DNP experiments were performed on frozen solutions of 10 mM biradical in d8-glycerol : D_2_O : H_2_O 60 : 30 : 10 v/v/v with 0.25 M U–^13^C–^15^N proline. DNP experiments at 800 MHz were performed on a Bruker BioSpin 527 GHz solid-state NMR DNP spectrometer. This spectrometer is equipped with a Bruker 800 WB/RS Plus magnet with a sweep coil, an Avance III NMR console, and a low-temperature 3.2 mm triple-resonance DNP MAS NMR probe.^45^ A gyrotron microwave source emits microwaves at a frequency of 527.043 GHz. In the DNP experiments the nuclear polarization is measured through the spectrum of ^13^C–^15^N proline, which is observed *via*^13^C–^1^H cross-polarization (CP). A CP spin-locking field of 50 kHz is applied on ^13^C, while the spin-locking field on ^1^H is ramped from 36 to 45 kHz. The contact time was set to 2 ms. During the acquisition SPINAL-64 decoupling^53^ was used at 83 kHz. Each CP acquisition was preceded by a presaturation sequence consisting of 300 90° ^1^H pulses 20 μs apart at an RF power of 100 kHz, followed by a period of polarization build-up, which was set to 1.26*T*_1_ for optimal sensitivity. Each spectrum was acquired with a 4-step phase cycle and acquired three times to confirm stability and reproducibility. The MAS frequency was set to 8 kHz and the sample temperature was kept at 103 K, unless noted otherwise. The enhancement, *ε*, was obtained from comparison of the ^13^C signal amplitude with and without microwaves. *T*_1_ (microwaves off) and *T*_B_ (microwaves on) were measured *via* a saturation recovery experiment. *T*_2_ was determined from the decay of the echo intensity during a rotor-synchronized Hahn echo sequence. To obtain the CE DNP enhancement field profiles the magnetic field is swept and at each field position the nuclear polarization is measured *via* the cross-polarization experiment as described above.

## Conflicts of interest

There are no conflicts to declare.

## Supplementary Material

Supplementary information
