# Supplementary material for: Diastereoisomers of l-proline-linked trityl-nitroxide biradicals: synthesis and effect of chiral configurations on exchange interactions
Source: Chem Sci. 2018 Apr 5;9(19):4381–91. doi: 10.1039/c8sc00969d (PMC5958346; doi:10.1039/c8sc00969d)
Supplement: Supplementary file 1 [file SC-009-C8SC00969D-s001.pdf]

Electronic Supplementary Material (ESI) for Chemical Science.
This journal is © The Royal Society of Chemistry 2018

Supporting Information

Diastereoisomers of L-Proline-linked trityl-nitroxide biradicals: synthesis and effect of chiral configurations on exchange interactions

Weixiang Zhai,[†] Yalan Feng,[†] Huiqiang Liu,[†] Antal Rockenbauer, [*,‡] Deni Mance,[§] Shaoyong Li, [†] Yuguang Song, [*,†] Marc Baldus,[§] Yangping Liu[*,†]

[†]Tianjin Key Laboratory on Technologies Enabling Development of Clinical Therapeutics and Diagnostics, School of Pharmacy, Tianjin Medical University, Tianjin 300070, P. R. China

[‡]Institute of Materials and Environmental Chemistry, Hungarian Academy of Sciences and, Department of Physics, Budapest University of Technology and Economics, Budafoki ut 8, 1111 Budapest, Hungary

[§]NMR Spectroscopy, Bijvoet Center for Biomolecular Research, Department of Chemistry, Faculty of Science, Utrecht University, 3584 CH Utrecht, The Netherlands

Contents

HPLC chromatograms of $TNT_{1,2}$ and $TNL_{1,2}$ 2

EPR spectra of NP_1 and NP_2 3

Reduction of four biradical diastereoisomers with ascorbic acid 4

ECD computational method 4

Temperature effect on the exchange interactions 12

Solvent effect on the exchange interactions....................13

MS and HRMS data....................15

HPLC chromatograms of $TNT_{1,2}$ and $TNL_{1,2}$

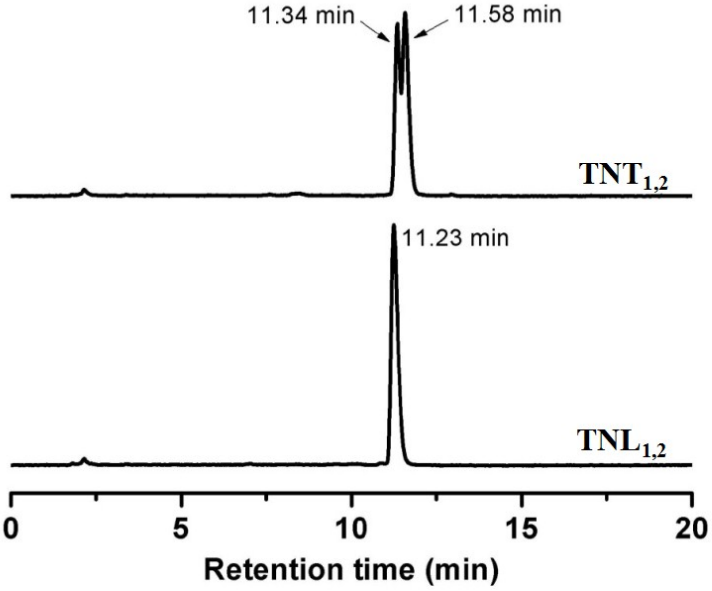

Figure S1. HPLC chromatograms of $TNT_{1,2}$ and $TNL_{1,2}$. The purities of $TNT_{1,2}$ and $TNL_{1,2}$ were determined to be 97% and 98%, respectively.

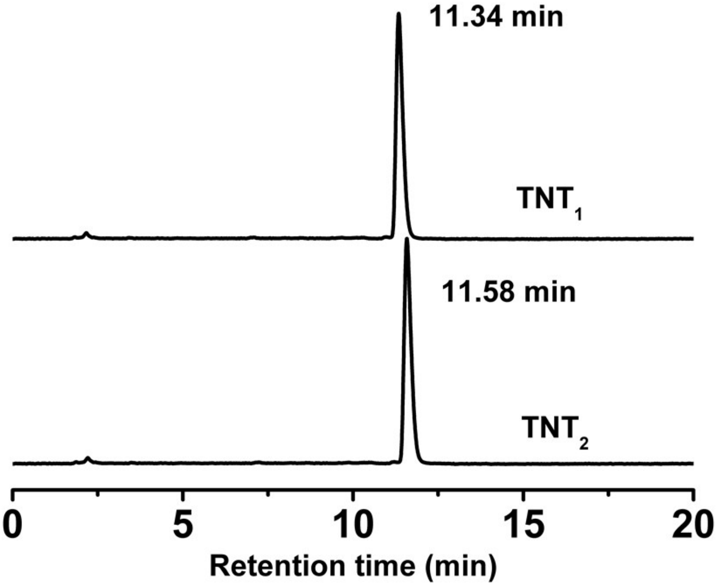

Figure S2. HPLC chromatograms of TNT_1 and TNT_2. The purities of TNT_1 and TNL_2 were determined to be ~ 98% for both of them. RP-HPLC: Retention time =11.34 min, 11.58min for TNT and 11.23min for TNL. Column: XBridge C18 5 μm 4.6 x 250 mm equipped with a guard column XBridge C18 5 μm 4.6 x 50mm. Column temperature: 25°C, UV detection at 254 nm.

Time	Flow	CH_3CN	NH_4OAc 20 mM
0	1 mL/min	30%	70%
20 min	1 mL/min	50%	50%

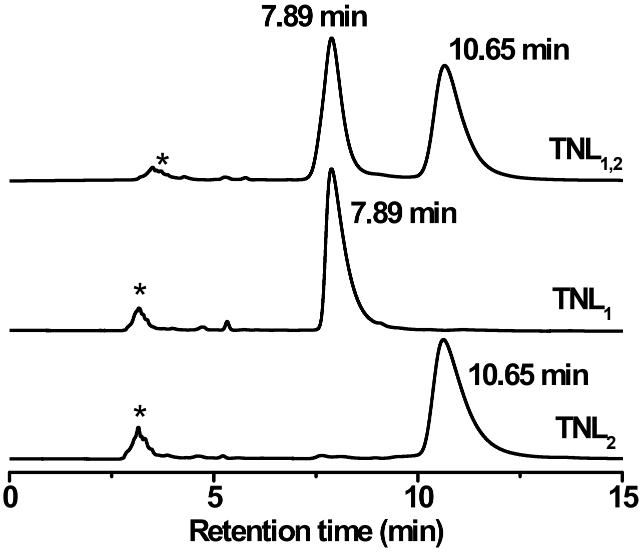

Figure S3. Chiral HPLC chromatograms of $TNL_{1,2}$. The purities of $TNT_{1,2}$ and $TNL_{1,2}$ were determined to be > 95%. Chiral HPLC: Retention time =7.89 min for TNL_1 and 10.65 min for TNL_2. Column: CHIRALPAK® IG (5μm, 1.6mm x 250mm). Column temperature: 25 ºC, UV detection at 254 nm, flow rate: 1 ml/min, eluent: ethanol/n-hexane 25%/75% (0.1% AcOH was added). *, peak from the solvent.

EPR spectra of NP_1 and NP_2

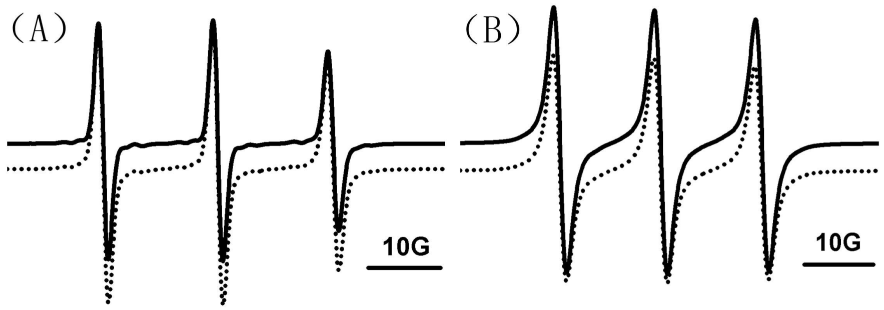

Figure S4. EPR spectra of NP_1 (solid line) and NP_2 (dotted line) in $MeOH/H_2O$ (v/v, 1:2) (A) and CH_2Cl_2 (B). α_N = 15.7 G in $MeOH/H_2O$ (v/v, 1:2) or 14.5 G in CH_2Cl_2 for NP_1. α_N = 15.7 G in $MeOH/H_2O$ (v/v, 1:2) or 14.5 G in CH_2Cl_2 for NP_2.

Reduction of four biradical diastereoisomers with ascorbic acid

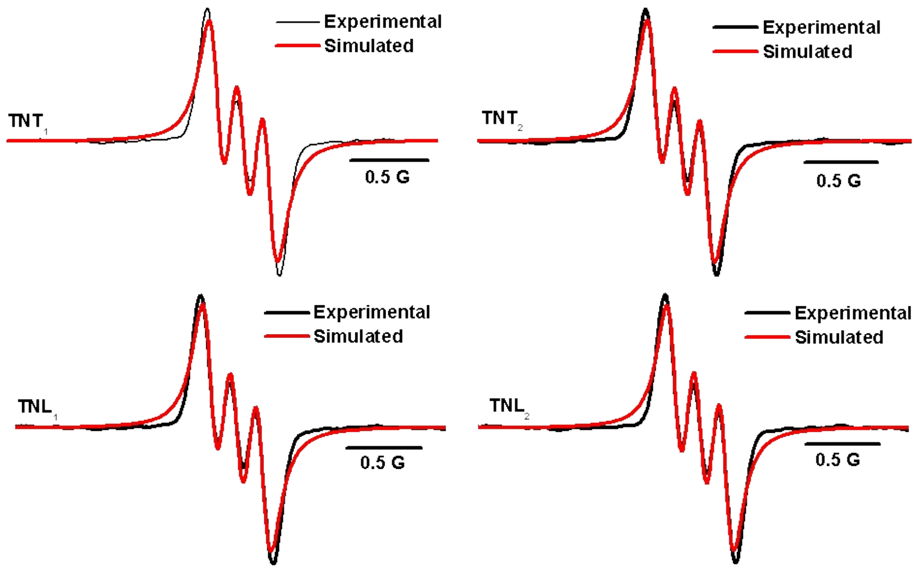

Figure S5. EPR spectra of trityl monoradicals obtained by reduction of four biradical diastereoisomers (50 μM) with ascorbic acid (2 mM) in phosphate buffer (20 mM, pH 7.4) at room temperature. The values of a_N were 0.180 G for TNT_1, 0.176 G for TNT_2, 0.180 G for TNL_1 and 0.176 G for TNL_2.

ECD computational method

A random conformational search of NP_1, TNT_2 and TNL_2 in Discovery studio 4.0 was used to produce low energy conformers within an energy of 10 kcal/mol. Conformational energy optimizations in GAUSSIAN 091 were subsequently executed using the DFT method at UCAM-B3LYP/TZVP level for NP2 and UB3LYP/6-31G level for TNT_2 and TNL_2. The TD-DFT calculations of their low-energy conformations within 0-2.5 kcal/mol were performed at the same level with 60 single excited states. Solvent effect was taken into account by using the polarizable continuum model (PCM). ECD simulation was executed using Boltzmann statistics, and their ECD spectra were generated in the program SpecDis 1.632 by applying Gaussian band shape with a 0.3, 0.18 and 0.20 eV exponential half-width for NP_2, TNT_2 and TNL_2, respectively. For all calculated spectra, the vertical axes were scaled to fit the experimental spectra. The wavelength shift of 3, -7 and -12 nm was employed for NP1, TNT_2 and TNL_2, respectively.

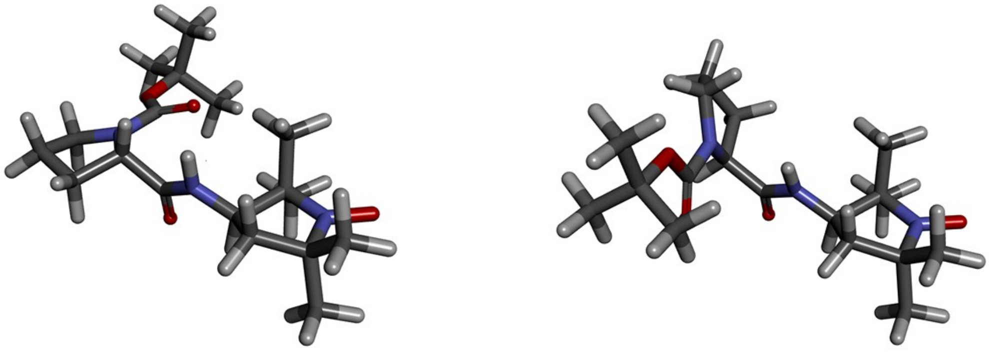

NP_1-1 **NP_1-2**

Figure S6. The most stable conformers of NP2 optimized at UCAM-B3LYP/TZVP level.

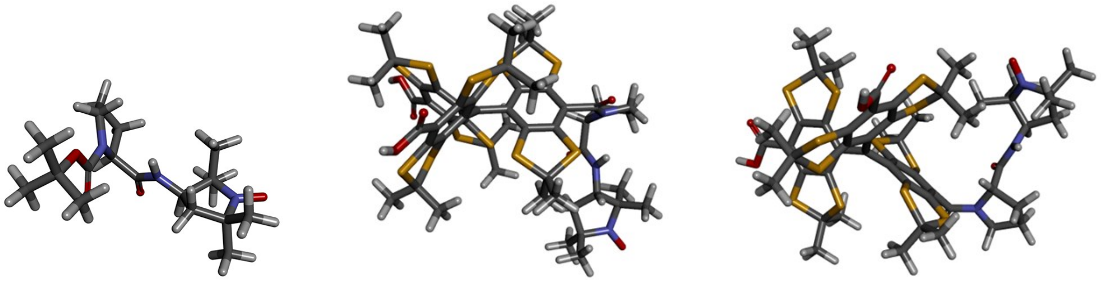

NP_1 **TNT_2** **TNL_2**

Figure S7 The absolute configurations of NP_1, TNT_2 and TNL_2 from the TD-DFT calculation.

Table S1. Relative free energies and Boltzmann populations of the most stable conformers of NP_2.

Conformers	Free energy (Hartree)	Free energy difference (Kcal/mol)	Boltzmann population (%)
NP_1-1	-1169.78132334	0	74
NP_1-2	-1169.78032619	0.62	26

Table S2. Cartesian coordinates of conformer NP_1-1 from UCAM-B3LYP/TZVP optimization.

atom	x	y	z		atom	x	y	z
C(1)	-1.481	3.455	-0.076		H(39)	6.394	-0.895	-0.844
C(2)	-3.000	3.580	-0.218		H(40)	5.894	-0.488	1.635
C(3)	-3.508	2.224	0.270		H(41)	5.964	1.248	1.266
N(4)	-2.436	1.316	-0.142		H(42)	4.550	0.572	2.096
C(5)	-1.192	1.997	-0.473		H(43)	2.142	-2.450	-1.526
C(6)	-0.020	1.425	0.326		H(44)	0.787	-1.300	-1.438
N(7)	1.109	1.208	-0.388		H(45)	2.324	-0.824	-2.200
C(8)	2.286	0.597	0.185		H(46)	1.884	-2.792	0.976
O(9)	-0.103	1.246	1.535		H(47)	0.609	-1.553	1.048
C(10)	3.592	1.148	-0.401		H(48)	2.048	-1.413	2.080
C(11)	4.658	0.106	-0.036		H(49)	-3.263	-2.284	1.990
N(12)	3.841	-1.127	0.060		H(50)	-3.581	-3.725	1.009
C(13)	2.378	-0.937	-0.013		H(51)	-2.189	-2.689	0.632
C(14)	5.729	-0.070	-1.108		H(52)	-6.181	-1.248	-0.099
C(15)	5.304	0.379	1.327		H(53)	-5.605	-1.482	1.564
C(16)	1.874	-1.399	-1.384		H(54)	-5.953	-2.888	0.538
C(17)	1.687	-1.724	1.095		H(55)	-4.434	-3.383	-1.458
O(18)	4.371	-2.277	0.179		H(56)	-4.663	-1.726	-2.042
O(19)	-3.747	-0.460	0.113		H(57)	-3.022	-2.353	-1.771
C(20)	-4.113	-1.875	0.044					
C(21)	-3.223	-2.692	0.975					
C(22)	-5.552	-1.871	0.543					
C(23)	-4.047	-2.362	-1.400					
C(24)	-2.532	-0.027	-0.259					
O(25)	-1.610	-0.732	-0.657					
H(26)	-0.924	4.164	-0.691					
H(27)	-1.190	3.599	0.969					
H(28)	-3.413	4.411	0.358					
H(29)	-3.273	3.724	-1.268					
H(30)	-4.455	1.928	-0.183					
H(31)	-3.629	2.206	1.360					
H(32)	-0.993	1.911	-1.548					
H(33)	1.055	1.280	-1.394					
H(34)	2.241	0.803	1.256					
H(35)	3.507	1.231	-1.490					
H(36)	3.829	2.139	-0.009					
H(37)	6.324	0.843	-1.198					
H(38)	5.277	-0.286	-2.081					

atom	x	y	z		atom	x	y	z
C(1)	1.205	3.358	-0.151		H(39)	-3.321	-3.755	-0.820
C(2)	2.469	3.334	-1.013		H(40)	-5.157	-2.226	1.251
C(3)	2.633	1.853	-1.357		H(41)	-3.716	-3.200	1.611
N(4)	2.085	1.175	-0.173		H(42)	-3.805	-1.512	2.149
C(5)	1.319	2.072	0.689		H(43)	-3.599	1.326	-2.309
C(6)	-0.054	1.549	1.094		H(44)	-2.044	1.967	-1.742
N(7)	-0.621	0.659	0.250		H(45)	-2.174	0.277	-2.266
C(8)	-1.917	0.067	0.485		H(46)	-4.600	2.226	-0.159
O(9)	-0.608	1.984	2.099		H(47)	-3.016	2.690	0.503
C(10)	-1.959	-1.425	0.133		H(48)	-4.064	1.579	1.403
C(11)	-3.451	-1.763	0.008		H(49)	4.209	-2.282	-2.254
N(12)	-4.017	-0.447	-0.372		H(50)	5.588	-1.387	-1.581
C(13)	-3.082	0.696	-0.318		H(51)	5.407	-3.121	-1.248
C(14)	-3.753	-2.786	-1.083		H(52)	5.675	-0.858	0.873
C(15)	-4.068	-2.201	1.340		H(53)	5.533	-2.583	1.251
C(16)	-2.693	1.088	-1.746		H(54)	4.390	-1.425	1.963
C(17)	-3.733	1.871	0.402		H(55)	3.489	-3.909	0.248
O(18)	-5.225	-0.315	-0.743		H(56)	2.401	-2.719	0.993
O(19)	3.400	-0.601	-0.486		H(57)	2.331	-3.023	-0.758
C(20)	4.049	-1.867	-0.134					
C(21)	4.863	-2.182	-1.383					
C(22)	4.964	-1.666	1.069					
C(23)	2.996	-2.943	0.107					
C(24)	2.579	0.024	0.368					
O(25)	2.268	-0.364	1.485					
H(26)	1.119	4.244	0.480					
H(27)	0.315	3.308	-0.787					
H(28)	2.388	3.962	-1.903					
H(29)	3.329	3.677	-0.430					
H(30)	3.670	1.558	-1.516					
H(31)	2.061	1.582	-2.251					
H(32)	1.857	2.258	1.623					
H(33)	-0.066	0.338	-0.531					
H(34)	-2.126	0.205	1.548					
H(35)	-1.455	-1.597	-0.825					
H(36)	-1.456	-2.036	0.886					
H(37)	-3.334	-2.469	-2.043					
H(38)	-4.833	-2.906	-1.199					

Table S3. Cartesian coordinates of conformer NP_1-2 from UCAM-B3LYP/TZVP optimization.

atom	x	y	z		atom	x	y	z
C(1)	-1.695	0.032	0.204		C(39)	3.323	-1.285	2.890
C(2)	-0.452	-0.324	0.902		C(40)	-2.846	5.599	-1.064
C(3)	-2.632	-1.021	-0.236		O(41)	-6.045	-3.923	-2.645
C(4)	-1.990	1.449	-0.092		O(42)	-5.992	-4.834	-0.600
C(5)	0.007	0.430	2.016		O(43)	-3.835	6.172	-0.352
C(6)	1.221	0.130	2.651		O(44)	-2.218	6.214	-1.910
C(7)	2.042	-0.907	2.183		N(45)	4.400	-0.455	2.792
C(8)	1.573	-1.703	1.130		O(46)	3.374	-2.333	3.539
C(9)	0.352	-1.425	0.495		C(47)	5.555	-0.678	3.698
C(10)	-3.268	2.009	0.181		C(48)	6.406	0.595	3.547
C(11)	-3.567	3.353	-0.107		C(49)	5.417	1.650	3.026
C(12)	-2.591	4.184	-0.701		C(50)	4.490	0.839	2.094
C(13)	-1.319	3.643	-0.993		C(51)	5.076	0.800	0.662
C(14)	-1.017	2.307	-0.677		O(52)	5.147	1.864	0.041
C(15)	-3.164	-1.032	-1.554		N(53)	5.489	-0.407	0.210
C(16)	-4.060	-2.027	-1.979		C(54)	6.123	-0.744	-1.065
C(17)	-4.501	-3.025	-1.073		C(55)	5.651	0.044	-2.303
C(18)	-3.968	-3.036	0.238		C(56)	6.700	-0.276	-3.389
C(19)	-3.056	-2.047	0.646		N(57)	7.904	-0.577	-2.560
S(20)	2.463	-3.134	0.563		C(58)	7.678	-0.637	-1.090
C(21)	1.651	-3.179	-1.107		C(59)	6.992	0.915	-4.310
S(22)	-0.073	-2.535	-0.827		C(60)	6.348	-1.522	-4.222
S(23)	-0.900	1.784	2.732		C(61)	8.235	0.643	-0.444
C(24)	-0.095	1.682	4.405		C(62)	8.370	-1.874	-0.510
S(25)	1.646	1.122	4.067		O(63)	9.064	-0.729	-3.075
S(26)	-0.048	4.562	-1.829		C(64)	2.398	-2.292	-2.106
C(27)	1.340	3.415	-1.374		C(65)	1.568	-4.629	-1.591
S(28)	0.574	1.734	-1.221		C(66)	-3.659	0.083	-5.364
S(29)	-4.526	1.115	1.059		C(67)	-1.980	-1.687	-4.682
C(30)	-5.926	2.202	0.521		C(68)	-0.067	3.078	5.033
S(31)	-5.195	3.900	0.360		C(69)	-0.819	0.670	5.298
S(32)	-4.450	-4.216	1.475		C(70)	2.369	3.397	-2.509
C(33)	-3.093	-3.808	2.682		C(71)	1.984	3.845	-0.051
S(34)	-2.636	-2.043	2.371		C(72)	-6.496	1.730	-0.821
S(35)	-2.729	0.197	-2.763		C(73)	-6.999	2.220	1.614
C(36)	-3.171	-0.832	-4.236		C(74)	-3.635	-3.948	4.109
S(37)	-4.572	-1.914	-3.683		C(75)	-1.888	-4.726	2.447
C(38)	-5.556	-4.013	-1.394		H(76)	-6.743	-4.601	-2.731

Table S4. Cartesian coordinates of conformer TNT_2 from UB3LYP/6-31G optimization.

atom	x	y	z		atom	x	y	z
H(77)	-3.955	7.080	-0.693		H(115)	0.450	3.798	4.395
H(78)	6.092	-1.590	3.424		H(116)	-0.318	0.603	6.270
H(79)	5.175	-0.811	4.716		H(117)	-0.834	-0.325	4.845
H(80)	6.870	0.884	4.493		H(118)	-1.854	0.993	5.462
H(81)	7.209	0.434	2.821		H(119)	3.191	2.720	-2.255
H(82)	5.892	2.480	2.500		H(120)	2.796	4.398	-2.640
H(83)	4.818	2.058	3.848		H(121)	1.923	3.080	-3.455
H(84)	3.509	1.305	2.001		H(122)	2.358	4.872	-0.141
H(85)	5.325	-1.185	0.837		H(123)	2.831	3.191	0.185
H(86)	5.879	-1.800	-1.223		H(124)	1.266	3.811	0.774
H(87)	5.640	1.111	-2.084		H(125)	-7.299	2.404	-1.141
H(88)	4.643	-0.250	-2.608		H(126)	-6.909	0.721	-0.719
H(89)	7.818	0.678	-4.987		H(127)	-5.726	1.713	-1.598
H(90)	6.107	1.151	-4.910		H(128)	-7.817	2.889	1.323
H(91)	7.262	1.803	-3.729		H(129)	-7.422	1.217	1.740
H(92)	6.083	-2.373	-3.585		H(130)	-6.595	2.556	2.572
H(93)	5.500	-1.309	-4.882		H(131)	-2.852	-3.700	4.834
H(94)	7.205	-1.811	-4.838		H(132)	-3.938	-4.984	4.292
H(95)	7.693	1.535	-0.769		H(133)	-4.494	-3.295	4.281
H(96)	8.175	0.582	0.647		H(134)	-1.089	-4.489	3.159
H(97)	9.288	0.749	-0.723		H(135)	-2.182	-5.772	2.593
H(98)	8.007	-2.791	-0.987		H(136)	-1.493	-4.614	1.433
H(99)	8.178	-1.943	0.566					
H(100)	9.451	-1.811	-0.666					
H(101)	2.463	-1.260	-1.749					
H(102)	3.413	-2.676	-2.258					
H(103)	1.879	-2.294	-3.070					
H(104)	1.057	-4.670	-2.559					
H(105)	2.576	-5.034	-1.731					
H(106)	1.029	-5.261	-0.881					
H(107)	-3.960	-0.518	-6.229					
H(108)	-2.847	0.743	-5.687					
H(109)	-4.507	0.696	-5.049					
H(110)	-2.267	-2.313	-5.535					
H(111)	-1.150	-1.041	-4.989					
H(112)	-1.632	-2.338	-3.875					
H(113)	0.437	3.041	6.005					
H(114)	-1.091	3.429	5.205					

Table S5. Cartesian coordinates of conformer TNL-2 from UB3LYP/6-31G optimization.

atom	x	y	z		atom	x	y	z
C(1)	-1.599	0.056	0.182		C(39)	2.944	-3.357	1.515
C(2)	-0.403	-0.740	0.488		C(40)	-1.823	5.737	1.447
C(3)	-2.759	-0.576	-0.485		O(41)	-6.681	-3.483	-1.958
C(4)	-1.655	1.493	0.526		O(42)	-6.886	-1.664	-3.249
C(5)	0.287	-0.598	1.724		O(43)	-2.605	6.076	2.490
C(6)	1.395	-1.403	2.040		O(44)	-1.183	6.567	0.823
C(7)	1.886	-2.351	1.127		N(45)	4.171	-2.935	1.906
C(8)	1.213	-2.507	-0.095		O(46)	2.639	-4.556	1.535
C(9)	0.097	-1.720	-0.418		C(47)	5.118	-3.916	2.477
C(10)	-2.771	2.041	1.217		C(48)	6.358	-3.081	2.850
C(11)	-2.845	3.408	1.542		C(49)	5.865	-1.622	2.819
C(12)	-1.801	4.282	1.164		C(50)	4.815	-1.641	1.686
C(13)	-0.693	3.760	0.460		C(51)	5.501	-1.594	0.298
C(14)	-0.612	2.388	0.165		O(52)	5.586	-2.585	-0.425
C(15)	-3.381	0.029	-1.607		N(53)	6.048	-0.389	-0.013
C(16)	-4.475	-0.574	-2.249		C(54)	6.948	-0.197	-1.150
C(17)	-5.022	-1.785	-1.759		C(55)	8.087	0.795	-0.804
C(18)	-4.411	-2.406	-0.642		C(56)	7.624	2.204	-1.249
C(19)	-3.305	-1.799	-0.017		N(57)	6.504	1.875	-2.179
S(20)	1.683	-3.748	-1.278		C(58)	6.278	0.422	-2.410
C(21)	0.862	-2.918	-2.721		C(59)	8.715	2.978	-2.004
S(22)	-0.628	-2.064	-2.006		C(60)	7.075	3.067	-0.100
S(23)	-0.206	0.539	3.002		C(61)	7.022	-0.014	-3.690
C(24)	0.590	-0.387	4.402		C(62)	4.785	0.139	-2.553
S(25)	2.098	-1.181	3.662		O(63)	5.943	2.771	-2.898
S(26)	0.619	4.780	-0.162		C(64)	1.795	-1.901	-3.382
C(27)	1.810	3.389	-0.474		C(65)	0.394	-3.984	-3.716
S(28)	0.769	1.897	-0.840		C(66)	-4.000	2.657	-4.554
S(29)	-4.069	1.027	1.879		C(67)	-2.605	0.569	-4.886
C(30)	-5.293	2.403	2.067		C(68)	1.021	0.611	5.480
S(31)	-4.281	3.906	2.468		C(69)	-0.357	-1.451	4.962
S(32)	-5.002	-3.901	0.123		C(70)	2.656	3.744	-1.701
C(33)	-3.503	-4.209	1.174		C(71)	2.683	3.146	0.762
S(34)	-2.767	-2.545	1.503		C(72)	-6.077	2.613	0.767
S(35)	-2.833	1.573	-2.295		C(73)	-6.222	2.093	3.244
C(36)	-3.584	1.306	-3.964		C(74)	-3.942	-4.841	2.499
S(37)	-5.106	0.276	-3.679		C(75)	-2.507	-5.105	0.427
C(38)	-6.261	-2.282	-2.400		H(76)	-7.517	-3.684	-2.422

atom	x	y	z		atom	x	y	z
H(77)	-2.588	7.050	2.567		H(115)	1.696	1.372	5.080
H(78)	5.338	-4.699	1.746		H(116)	0.138	-2.006	5.767

H(79)	4.648	-4.390	3.345	H(117)	-0.663	-2.162	4.189
H(80)	6.762	-3.363	3.825	H(118)	-1.255	-0.973	5.370
H(81)	7.149	-3.227	2.108	H(119)	3.392	2.962	-1.910
H(82)	6.668	-0.901	2.645	H(120)	3.213	4.668	-1.509
H(83)	5.364	-1.357	3.756	H(121)	2.034	3.892	-2.588
H(84)	4.101	-0.818	1.769	H(122)	3.264	4.047	0.991
H(85)	5.925	0.375	0.637	H(123)	3.381	2.322	0.574
H(86)	7.337	-1.187	-1.391	H(124)	2.075	2.893	1.636
H(87)	8.987	0.508	-1.354	H(125)	-6.772	3.453	0.884
H(88)	8.338	0.759	0.260	H(126)	-6.655	1.714	0.528
H(89)	8.311	3.916	-2.394	H(127)	-5.409	2.830	-0.071
H(90)	9.546	3.209	-1.329	H(128)	-6.930	2.917	3.388
H(91)	9.105	2.393	-2.843	H(129)	-6.805	1.191	3.032
H(92)	6.308	2.544	0.481	H(130)	-5.664	1.943	4.172
H(93)	7.885	3.350	0.581	H(131)	-3.068	-5.015	3.137
H(94)	6.625	3.978	-0.505	H(132)	-4.412	-5.812	2.311
H(95)	8.107	0.095	-3.593	H(133)	-4.650	-4.205	3.036
H(96)	6.803	-1.063	-3.916	H(134)	-1.621	-5.277	1.048
H(97)	6.690	0.601	-4.533	H(135)	-2.971	-6.073	0.206
H(98)	4.231	0.476	-1.673	H(136)	-2.188	-4.648	-0.514
H(99)	4.624	-0.935	-2.674				
H(100)	4.392	0.657	-3.432				
H(101)	2.116	-1.137	-2.670				
H(102)	2.684	-2.409	-3.774				
H(103)	1.281	-1.407	-4.214				
H(104)	-0.130	-3.512	-4.554				
H(105)	1.260	-4.516	-4.125				
H(106)	-0.275	-4.710	-3.247				
H(107)	-4.481	2.507	-5.527				
H(108)	-3.116	3.285	-4.716				
H(109)	-4.693	3.188	-3.897				
H(110)	-3.074	0.385	-5.859				
H(111)	-1.706	1.176	-5.042				
H(112)	-2.303	-0.391	-4.458				
H(113)	1.526	0.084	6.297				
H(114)	0.140	1.108	5.901				

Table S6. Parameters obtained by simulating EPR spectra of TNT_1, TNT_2, TNL_1 and TNL_2 in phosphate buffer (20 mM, pH 7.4) at room temperature.

Biradical	g (G)	A_N (G)	J (G)	B (G)	γ (G)
TNT_1	2.0046	16.0	252	-0.01	-0.12
TNT_2	2.0046	16.0	128	-0.05	-0.25
TNL_1	2.0044	15.7	14	-0.78	-0.35
TNL_2	2.0045	15.5	31	-0.61	-0.50

Temperature effect on the exchange interactions

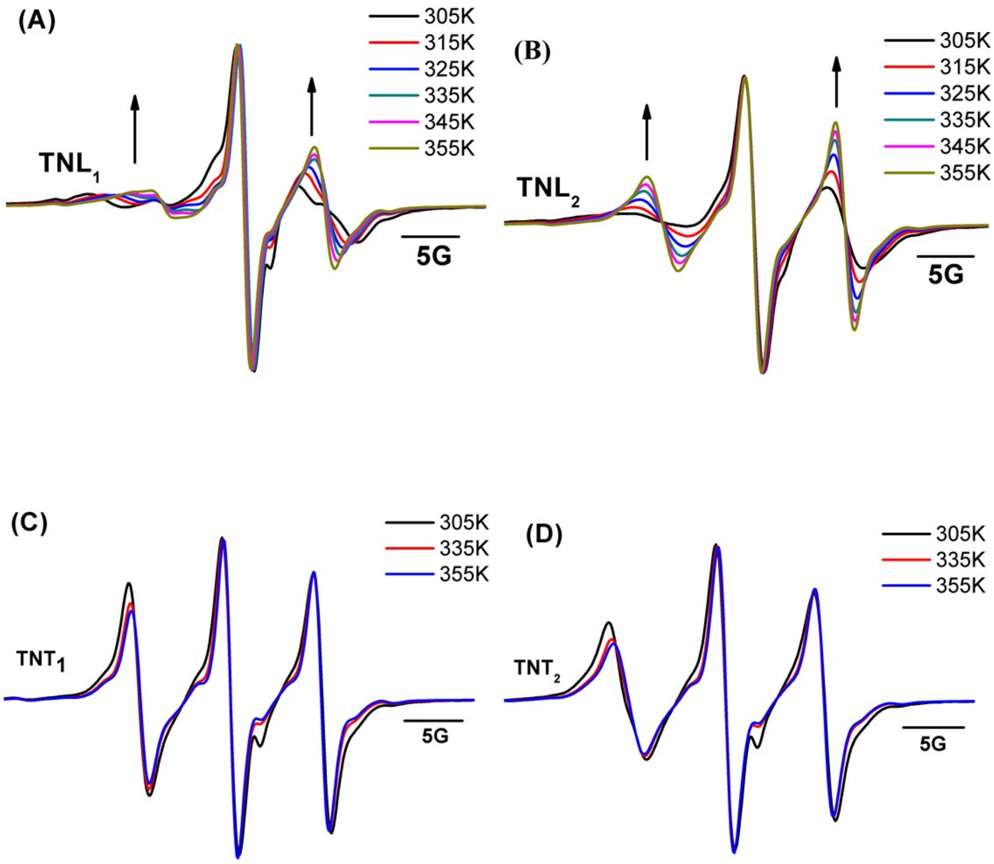

Figure S8. Normalized EPR spectra of TNL_1 (A), TNL_2 (B), TNT_1 (C) and TNL_2 (D) in PBS (20 mM, pH 7.4) at different temperatures.

Solvent effect on the exchange interactions

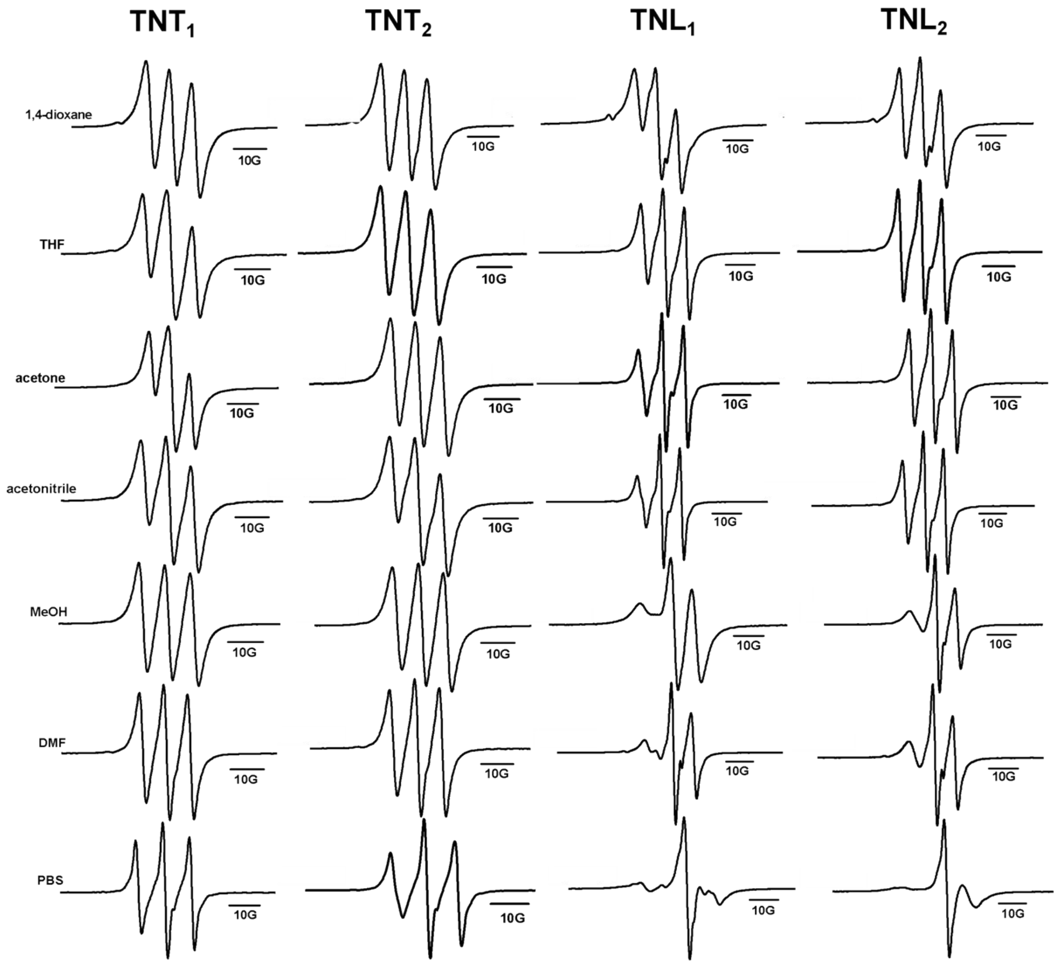

Figure S9. EPR spectra of TNT_1, TNT_2, TNL_1 and TNL_2 in different solvents at room temperature.

Table S7. Parameters obtained by simulating EPR spectra of TNT_1, TNT_2, TNL_1 and TNL_2 in

glycerol/water (v/v, 60/40) at low temperatures (~220K).

Sample	g_{xx}	g_{yy}	g_{zz}	A_{xx}	A_{yy}	A_{zz}	*J*	*D*
TNT_1	2.007	1.999	2.003	7.425	2.754	35.503	195.7	9.2
TNT_2	2.0020	1.996	1.999	9.646	5.365	33.400	172.4	11.3
TNL_1	2.007	2.007	2.002	3.995	9.911	35.109	5.3	7.7
TNL_2	2.008	2.006	2.003	10.640	8.717	34.381	6.3	7.7

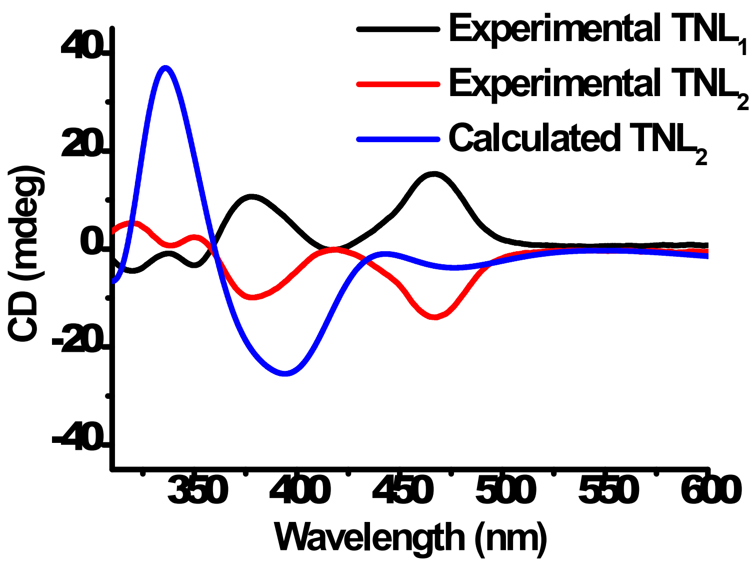

Figure S10 Comparison of calculated ECD spectra of TNL_2 with the experimental ones of TNL_1 and TNL_2.

MS and HRMS data

Display Report

Analysis Info

Analysis Name	D:\Data\lhq\cto2-NN-1.d	Acquisition Date	11/2/2015 10:33:30 AM
Method	full scan.m	Operator	BDAI
Sample Name	cto2-NN-1	Instrument	HCT
Comment			

Acquisition Parameter

Ion Source Type	ESI	Ion Polarity	Positive	Alternating Ion Polarity	on
Mass Range Mode	Ultra Scan	Scan Begin	50 m/z	Scan End	800 m/z
Capillary Exit	117.6 Volt	Skimmer	40.0 Volt	Trap Drive	37.3
Accumulation Time	674 µs	Averages	5 Spectra	Auto MS/MS	off

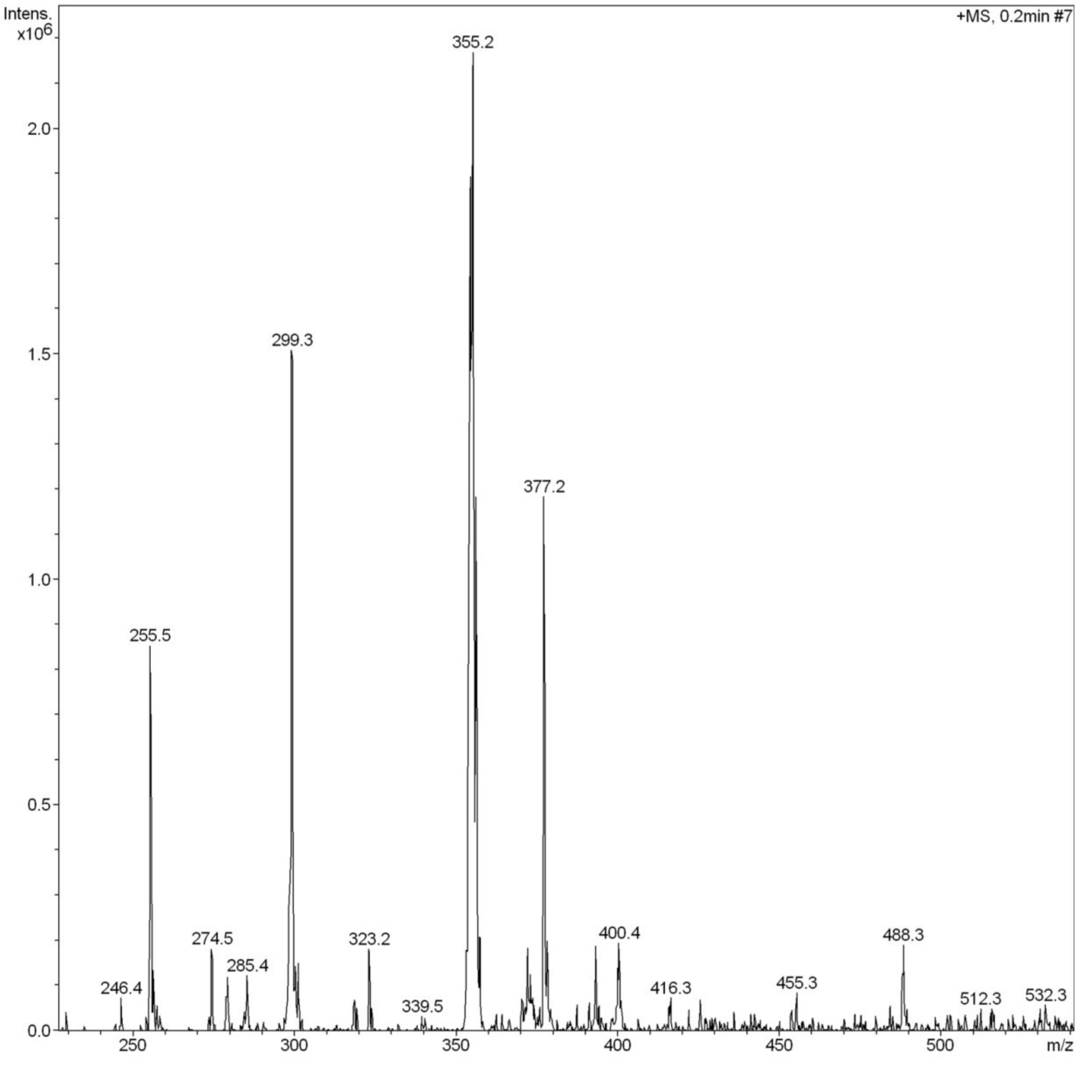

Bruker Compass DataAnalysis 4.0 printed: 1/26/2016 10:39:27 PM Page 1 of 1

Figure S11 HRMS spectrum of NP_1.

Display Report

Analysis Info

Analysis Name	D:\Data\lhq\cto2-NN-2.d	Acquisition Date	11/2/2015 10:43:14 AM
Method	full scan.m	Operator	BDAl
Sample Name	cto2-NN-2	Instrument	HCT
Comment			

Acquisition Parameter

Ion Source Type	ESI	Ion Polarity	Negative	Alternating Ion Polarity	on
Mass Range Mode	Ultra Scan	Scan Begin	50 m/z	Scan End	800 m/z
Capillary Exit	-117.6 Volt	Skimmer	-40.0 Volt	Trap Drive	50.3
Accumulation Time	200000 µs	Averages	5 Spectra	Auto MS/MS	off

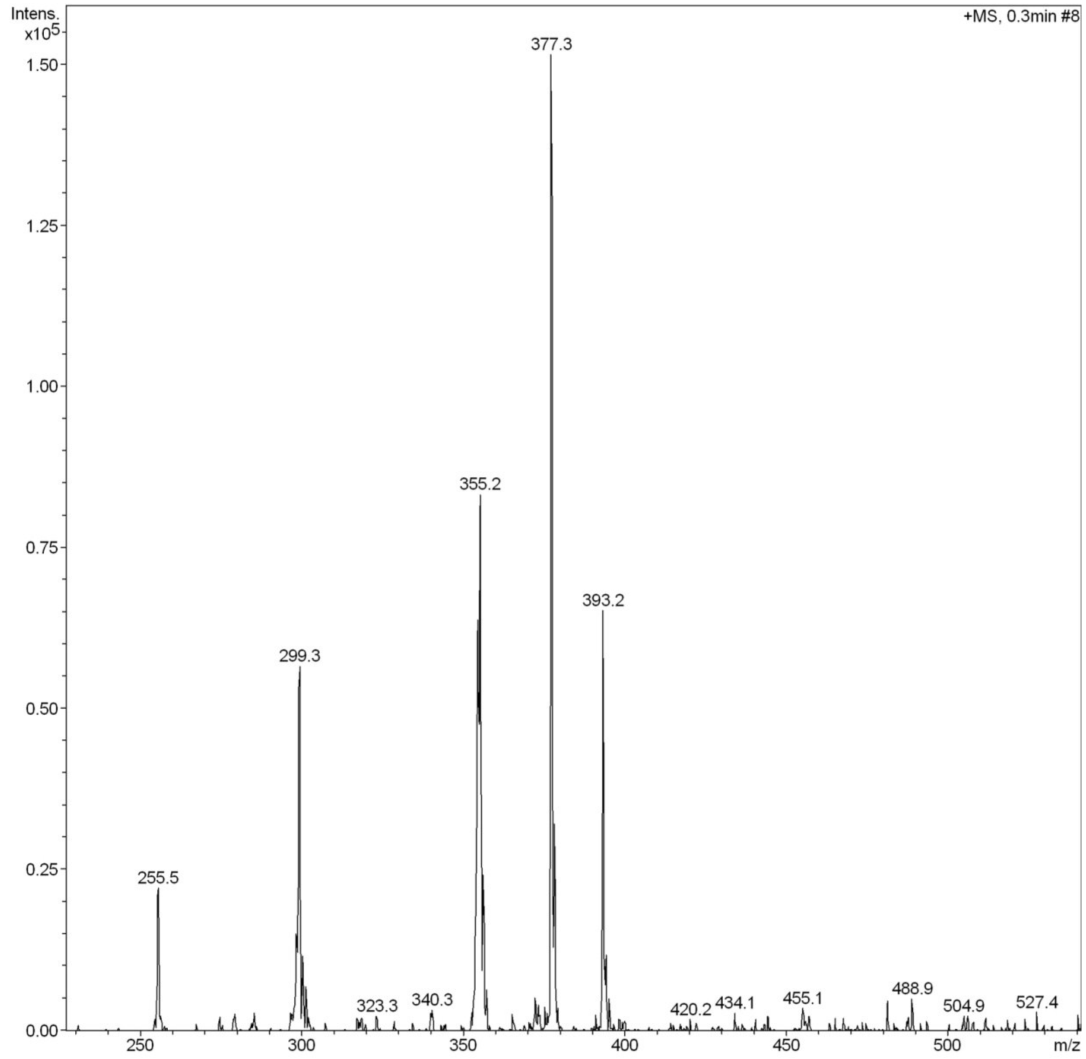

Bruker Compass DataAnalysis 4.0 printed: 1/26/2016 10:40:16 PM Page 1 of 1

Figure S12 HRMS spectrum of NP_2.

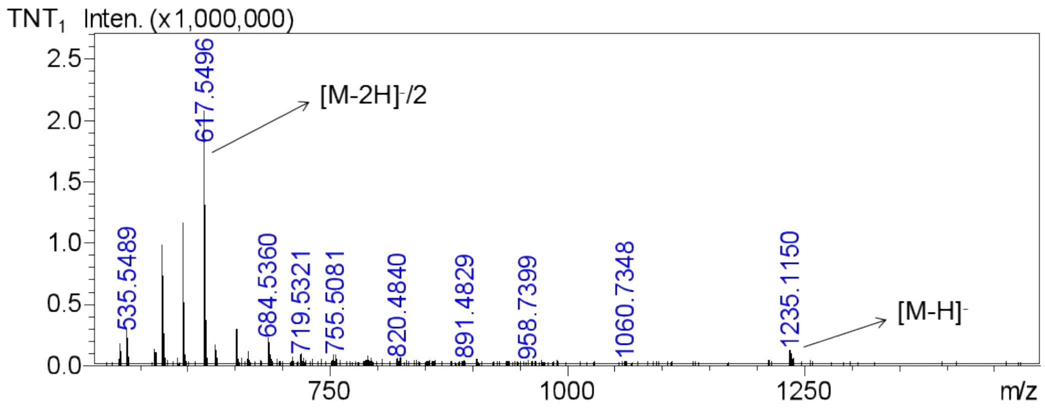

Figure S13 HRMS spectrum of TNT_1.

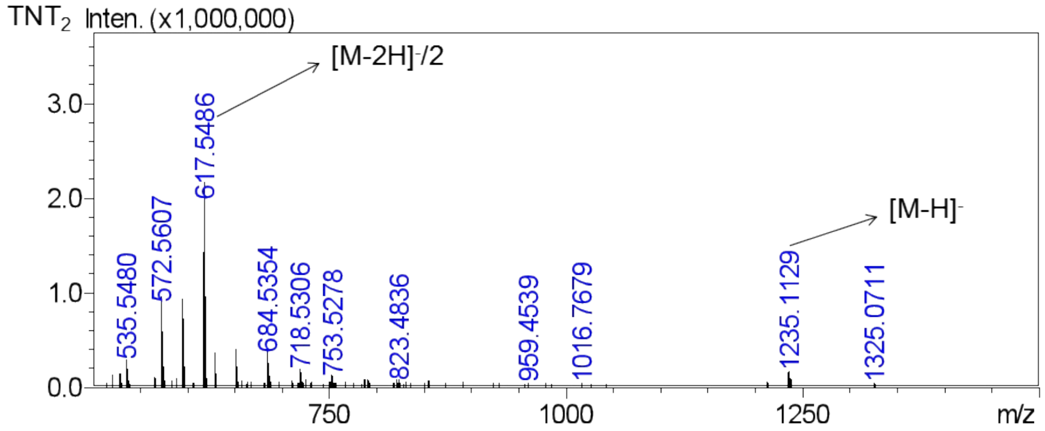

Figure S14 HRMS spectrum of TNT_2.

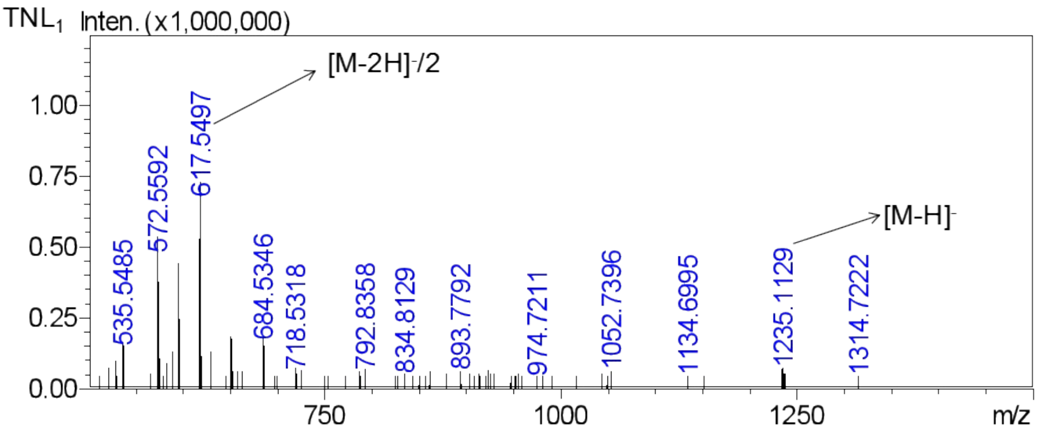

Figure S15 HRMS spectrum of TNL$_1$.

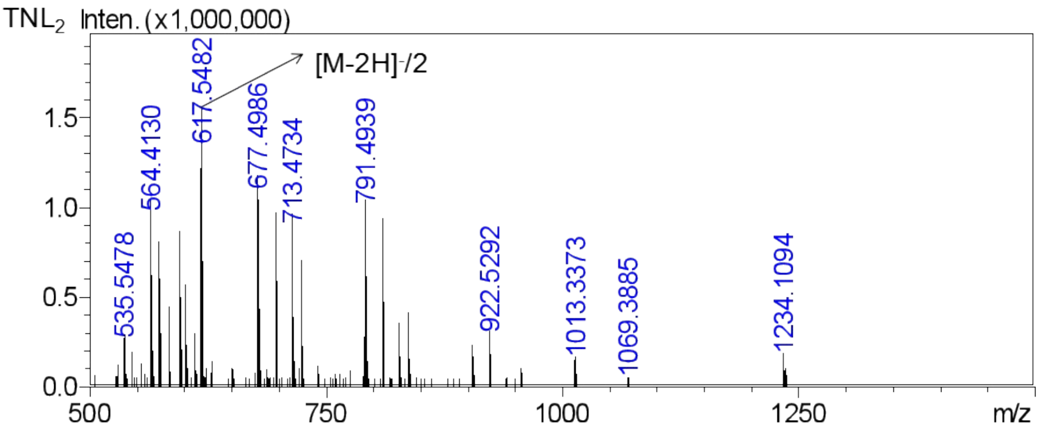

Figure S16 HRMS spectrum of TNL$_2$.
